# A Two-Stage Screening-to-Optimization Approach with Mechanistic Model Analysis: Enhancing Anthocyanin in Lettuce Without Yield Loss

**DOI:** 10.3390/plants15050838

**Published:** 2026-03-09

**Authors:** Zhihao Wei, Wei Fang, Chen-Kang Huang

**Affiliations:** Department of Biomechatronics Engineering, National Taiwan University, Taipei 10617, Taiwan; d02631007@ntu.edu.tw

**Keywords:** UV-A radiation, red-leaf lettuce, anthocyanin biosynthesis, controlled-environment agriculture, mechanistic modeling, carbon competition, growth–defense tradeoff

## Abstract

Enhancing anthocyanin accumulation in red-leaf lettuce grown in plant factories often incurs yield penalties. Here we propose a two-stage screening-to-optimization framework integrated with mechanistic modeling to resolve this tradeoff. In Stage 1, comparative experiments confirmed that UV-A is more compatible with growth and pigmentation than UV-B, and identified ‘Lollo Rosso’ as a highly responsive cultivar. In Stage 2, optimization experiments showed that L6D6 (6 h day^−1^ for 6 days) increased the total anthocyanin per plant by 19.9% while maintaining fresh weight. Motivated by observed nonlinear phenomena including biomass overcompensation, circadian disruption under night irradiation, and ontogeny-dependent vulnerability, we developed a six-state ordinary differential equation (ODE) model that integrates reactive oxygen species (ROS) dynamics with stress damage–repair processes. A key innovation is the explicit representation of carbon competition between growth and antioxidant defense, where AOX synthesis consumes carbon from the buffer pool, creating a physiologically meaningful growth–defense tradeoff supported by the Growth-Differentiation Balance Hypothesis. The model achieved high accuracy in an independent validation set that included extreme doses (errors ≤ 10.6%, with 11 of 12 metrics < 10%), supporting the physiological necessity of the introduced mechanisms. Global optimization based on the calibrated model predicted that 9 h day^−1^ for 4 days is the theoretical optimum, potentially increasing total anthocyanin by 38.3% with minimal fresh-weight reduction (−2.4%), substantially outperforming the best experimental treatment. This quantitative mechanistic framework provides a scientific basis for designing precise stress-light recipes in controlled-environment agriculture.

## 1. Introduction

Lettuce (*Lactuca sativa* L.) is widely consumed, and red-leaf lettuce is distinguished by its high anthocyanin content. Dietary supplementation with red-pigmented lettuce has been reported to improve lipid profiles and antioxidant status in a mouse model, suggesting potential cardiovascular benefits [1]. Light is a key signal regulating plant development and metabolism across the life cycle [2]. Plants perceive light cues through photoreceptors such as phytochromes, cryptochromes, and UVR8 [3], which activate transcription factors including HY5 to induce the expression of anthocyanin biosynthetic genes [4,5]. In subtropical plant factories, however, artificial lighting typically lacks ultraviolet bands, which may limit the normal pigmentation of red-leaf lettuce.

The coloration of plants and their synthesis of secondary metabolites are primarily defense mechanisms against environmental stresses, with solar UV radiation being a direct and effective stressor [6,7]. Numerous studies have confirmed that supplemental UV radiation is an effective tool for inducing the accumulation of anthocyanins and other secondary metabolites in plants [8]. However, the application of stress is a double-edged sword; while it can enhance quality, it may also inhibit plant growth. Krizek et al. [9] demonstrated that solar UV-A and UV-B radiation, while inducing anthocyanin production, also significantly decreased the fresh and dry weight of red lettuce (cv. ‘New Red Fire’). This growth–defense tradeoff has been extensively studied in ecological literature. The Growth-Differentiation Balance Hypothesis [10] posits that plants allocate limited carbon resources between growth and defense, creating an inherent tradeoff. Recent work has further elucidated the coordinated resource allocation mechanisms underlying this tradeoff [11]. Understanding the carbon costs of defense compound synthesis [12,13] is crucial for optimizing light recipes that balance yield and quality.

This highlights the complexity of UV application, which is contingent on wavelength specificity. The distinct roles of different UV wavebands are critical. Weiland et al. [14] noted that supplemental UV-B can increase flavonoid and anthocyanin concentrations, but its higher photon energy readily leads to significant growth inhibition. In contrast, UV-A is considered a more moderate and controllable regulatory tool [15]. This aligns with findings from Li and Kubota [16], who reported that UV-A and blue light could increase the anthocyanin content of ‘Red Cross’ lettuce. Chen et al. [17] further observed that UV-A radiation is beneficial for yield and quality of indoor cultivated lettuce, demonstrating that UV-A can even increase biomass. Similarly, Lee et al. [18] found that brief pre-harvest exposure to supplemental UV-A LEDs enhanced the nutritional quality of lettuce without compromising growth. Recognizing these advantages, Lycoskoufis et al. [19] developed a strategy involving a UV-blocking film supplemented with UV light for 10 days before harvest to increase fresh weight by 30% while maintaining equivalent quality, demonstrating the practical potential of managed UV supplementation in greenhouse production.

Beyond wavelength, the plant’s response is also governed by genotypic specificity. Garcia-Macias et al. [20] studied ‘Lollo Rosso’ lettuce under plastic films varying in ultraviolet transparency and discovered that UV irradiation induced a substantial accumulation of anthocyanins and phenolic acids. This suggests that responses can be cultivar-specific, a factor that must be considered when developing UV supplementation strategies for controlled-environment agriculture.

Regarding crop growth models, existing lettuce models [21,22] focus primarily on dry matter accumulation under non-stress conditions and do not explicitly represent the dynamic impact of UV stress on yield and quality. There is still a lack of a quantitative mechanistic model that captures UV-induced stress, damage–repair dynamics, carbon competition between growth and defense, and their interactive effects on biomass and anthocyanin. Based on the foregoing literature, it is evident that a one-size-fits-all approach is inadequate for leveraging UV light to enhance crop quality; a systematic framework is required to navigate the complexities of wavelength and genotype specificity. Therefore, this study aims to develop and validate a two-stage screening-to-optimization approach integrating experiments and mechanistic modeling: (1) Screening stage: compare UV-A and UV-B across multiple cultivars to identify a safe, effective waveband and a resilient, responsive cultivar; (2) Optimization stage: subject the selected candidate to various UV-A irradiation protocols to identify a strategy that enhances coloration and secondary metabolites without compromising fresh weight; and (3) Mechanistic integration: develop a continuous, physiologically interpretable model that integrates stress damage–repair dynamics, carbon competition, ontogeny-dependent vulnerability, and UV-A morphological effects, and calibrate parameters using data-driven fitting to enable quantitative prediction and recipe optimization.

## 2. Results

### 2.1. Experiment 1: Cultivar Screening Under UV-A and UV-B

**Figure 1:**
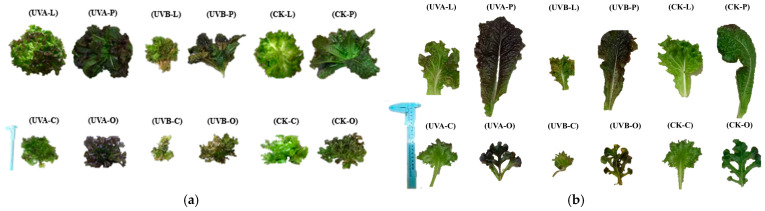
Morphology of four lettuce cultivars at 35 DAS under control (no UV), UV-A, and UV-B. (**a**) Whole-plant representative images; (**b**) close-up of the central leaves. UV-B caused clear necrosis, whereas UV-A produced uniform pigmentation without injury.

**Figure 2:**
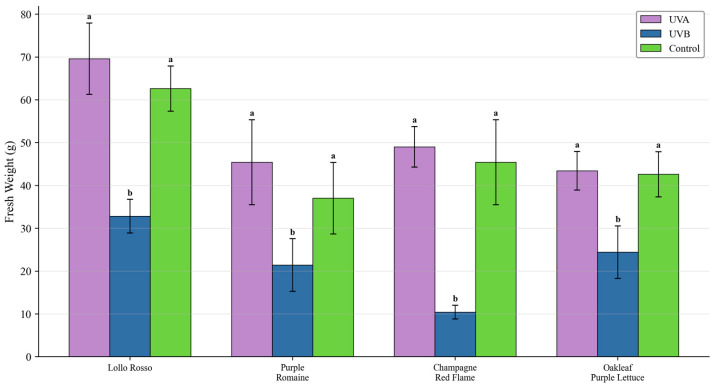
Effects of UV-A and UV-B on shoot fresh weight for four lettuce cultivars. Data are mean ± SD (n = 5). Different letters indicate significant differences (*p* < 0.05).

UV-B caused visible necrotic lesions and significant fresh-weight reduction in all cultivars, indicating severe photodamage. In contrast, UV-A induced red pigmentation without visible injury, and fresh weight was not statistically different from controls across cultivars. Representative phenotypes are shown in Figure 1, and fresh-weight responses are summarized in Figure 2.

#### Color Parameters and Anthocyanin Content

**Figure 3:**
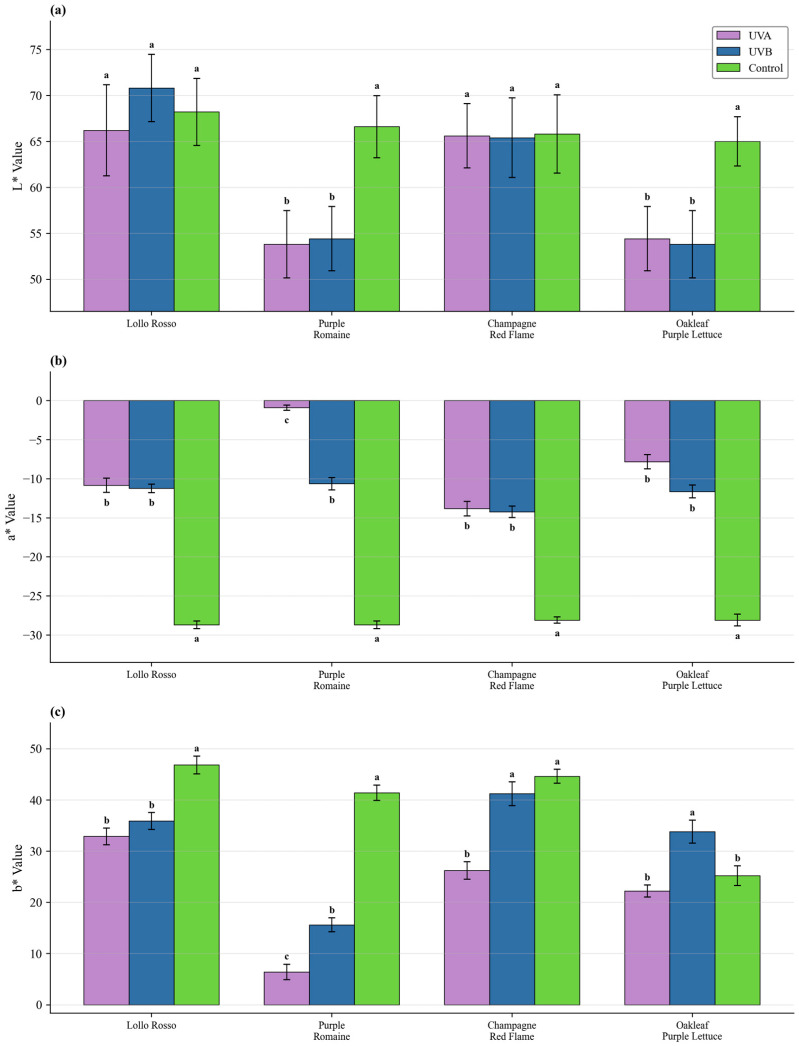
Effects of UV-A and UV-B on leaf color parameters (L*, a*, b*) across cultivars. UV treatments increased a* values indicating enhanced redness. (**a**) L*, (**b**) a*, (**c**) b*. Data are mean ± SD (n = 5). Different letters indicate significant differences (*p* < 0.05).

**Figure 4:**
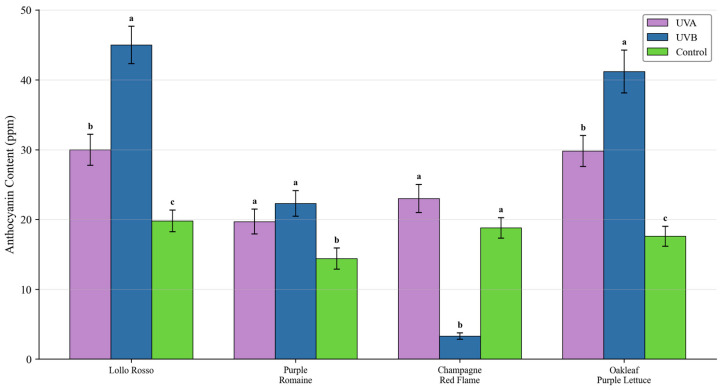
Effects of UV-A and UV-B on anthocyanin concentration across cultivars. UV-B induced the highest concentration but with strong growth inhibition. Data are mean ± SD (n = 5). Different letters indicate significant differences (*p* < 0.05).

Both UV-A and UV-B significantly decreased L* (lightness) and increased a* (redness). Although UV-B produced the highest anthocyanin concentration, it was accompanied by strong biomass penalties. Considering the growth-quality tradeoff, ‘Lollo Rosso’ showed robust growth and strong pigmentation response and was selected for optimization. Leaf color parameters are reported in Figure 3, and anthocyanin concentration is shown in Figure 4.

### 2.2. Experiment 2: Optimization of UV-A Recipes for ‘Lollo Rosso’

#### 2.2.1. Morphology and Fresh Weight

**Figure 5:**
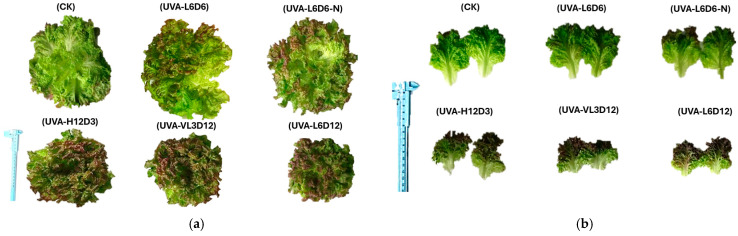
Morphology of ‘Lollo Rosso’ at 35 DAS under different UV-A optimization recipes. L6D6 produced compact plants with strong pigmentation. (**a**) Whole-plant representative images; (**b**) close-up of the central leaves.

**Figure 6:**
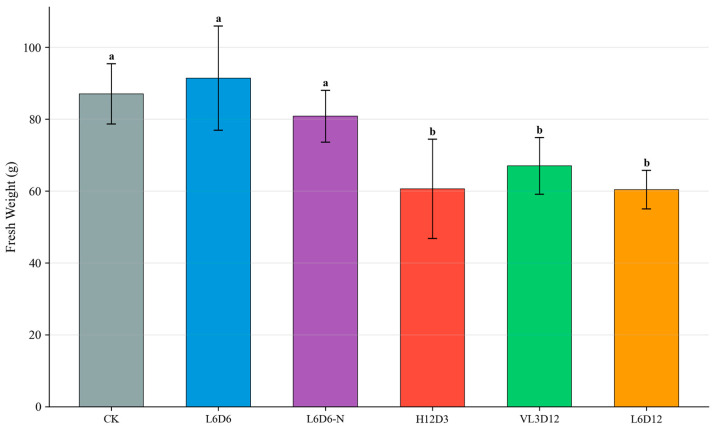
Effects of different UV-A recipes on shoot fresh weight of ‘Lollo Rosso’. Data are mean ± SD (n = 5). Different letters indicate significant differences (*p* < 0.05).

All UV-A treatments enhanced red coloration. Fresh weight was maintained or slightly increased only in L6D6 (91.4 g) compared with CK (87.0 g). Other recipes (H12D3, VL3D12, L6D12) significantly reduced fresh weight, indicating that higher daily dose or longer cumulative exposure is detrimental to growth. Representative phenotypes are shown in Figure 5, and fresh-weight responses are summarized in Figure 6.

#### 2.2.2. Color Parameters and Anthocyanin

**Figure 7:**
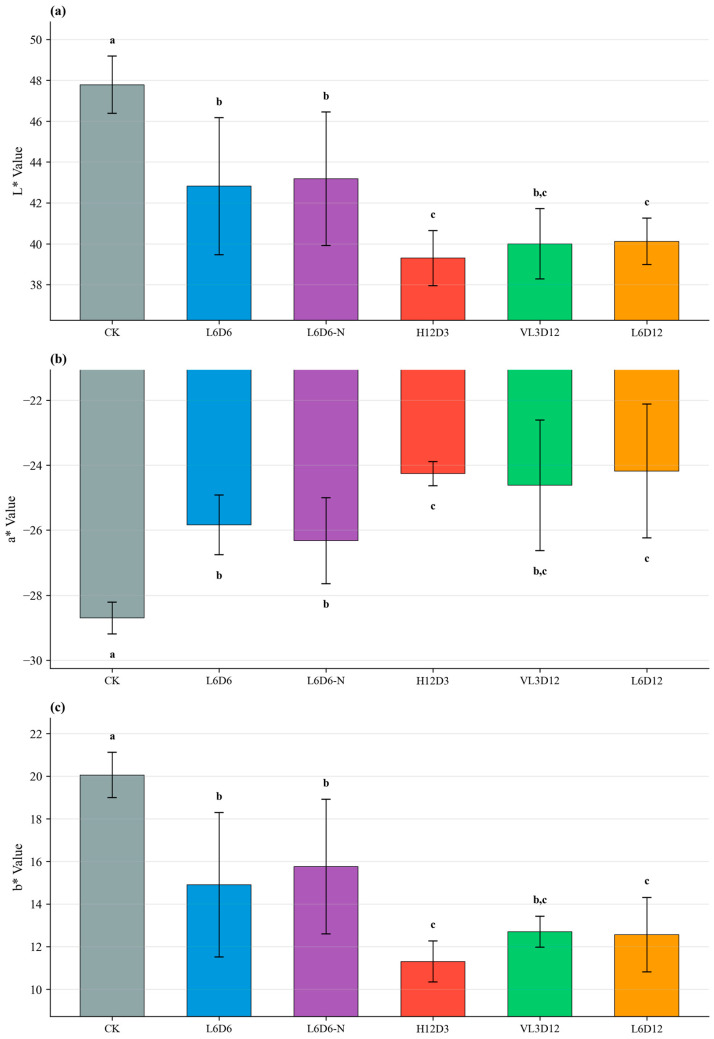
Effects of UV-A recipes on leaf color parameters (L*, a*, b*) of ‘Lollo Rosso’. (**a**) L*, (**b**) a*, (**c**) b*. Data are mean ± SD (n = 5). Different letters indicate significant differences (*p* < 0.05).

**Figure 8:**
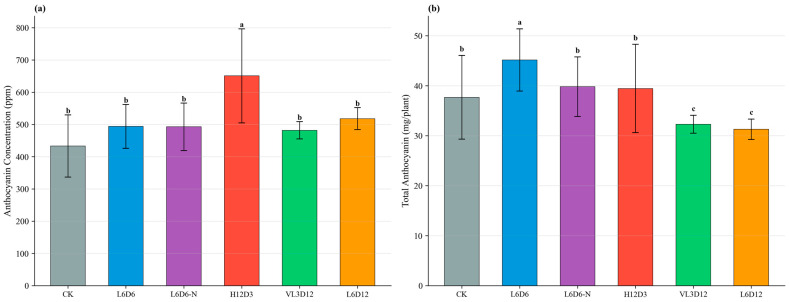
Effects of UV-A recipes on anthocyanin of ‘Lollo Rosso’: (**a**) concentration (ppm); (**b**) total anthocyanin per plant (mg), calculated as ppm × FW (kg), i.e., concentration (mg kg^−1^) × fresh weight (kg). Data are mean ± SD (n = 5). Different letters indicate significant differences (*p* < 0.05).

All UV-A treatments significantly increased a* values. H12D3 produced the highest anthocyanin concentration (651 ppm), but due to low biomass its per-plant total anthocyanin was not optimal. L6D6 combined higher biomass with elevated concentration (494 ppm), resulting in the highest anthocyanin amount per plant among tested recipes. Leaf color parameters are shown in Figure 7, and anthocyanin concentration and per-plant anthocyanin are shown in Figure 8.

### 2.3. Mechanistic Modeling Results

#### 2.3.1. Training-Set Calibration

The model fit the six training treatments with low errors (Table 1). Mean absolute errors were 2.3% for fresh weight (max 5.6%) and 2.4% for anthocyanin (max 5.6%), meeting the <5% target across 10 of 12 metrics. The carbon competition mechanism successfully captured the growth–defense tradeoff, with VL3D12 and L6D12 showing elevated stress levels and corresponding growth penalties.

#### 2.3.2. Independent Validation (3-Day Gradient)

**Figure 9:**
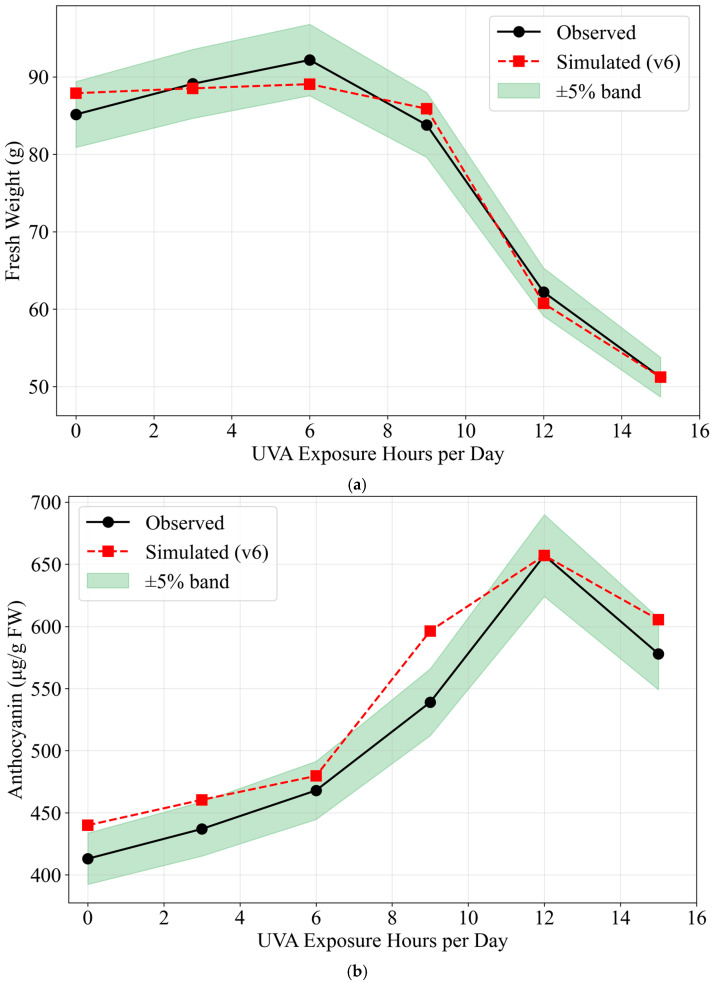

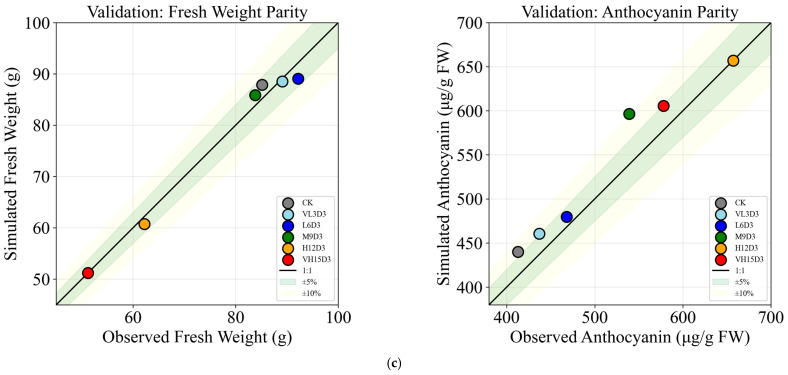
Validation set results. (**a**) Fresh-weight response vs. daily UV-A hours (0–15 h day^−1^). Blue circles represent observed values (mean ± SD, n = 5); the solid line shows model predictions. Fresh weight shows a biphasic response: slight increase at low doses (0–6 h day^−1^) due to UV-A morphological benefits, then decline at higher doses (≥9 h day^−1^) due to stress-induced growth inhibition. (**b**) Anthocyanin response vs. daily UV-A hours (0–15 h day^−1^), showing hormesis at the highest dose. Anthocyanin concentration peaks at 12 h day^−1^ (657 ppm) and declines at 15 h day^−1^ (578 ppm), demonstrating the hormesis phenomenon where excessive stress inhibits AOX synthesis efficiency. (**c**) Model prediction vs. observation with ±5% and ±10% error bands: fresh weight (**left**) and anthocyanin (**right**) parity plots. Points near the diagonal indicate good model fit; 11 of 12 metrics fall within ±10% error.

An independent 3-day gradient dataset was used to test generalization (Table 2). The validation set was conducted in a separate experimental batch from the training set; hence H12D3_val (validation) shows slightly different values from H12D3 (training: FW 60.6 g, Anth 651 ppm vs. validation: FW 62.2 g, Anth 657 ppm), reflecting normal batch-to-batch variation. The model achieved <10% errors for fresh weight across all validation treatments and <10% errors for anthocyanin in 5 of 6 treatments. Fresh-weight response, anthocyanin response, and prediction–observation parity are shown in Figure 9.

The model reproduced the non-monotonic anthocyanin response: 12 h day^−1^ produced the peak concentration (657 ppm) while 15 h day^−1^ declined (578 ppm), consistent with hormesis [23,24].

#### 2.3.3. Mechanistic Diagnostics

**Figure 10:**
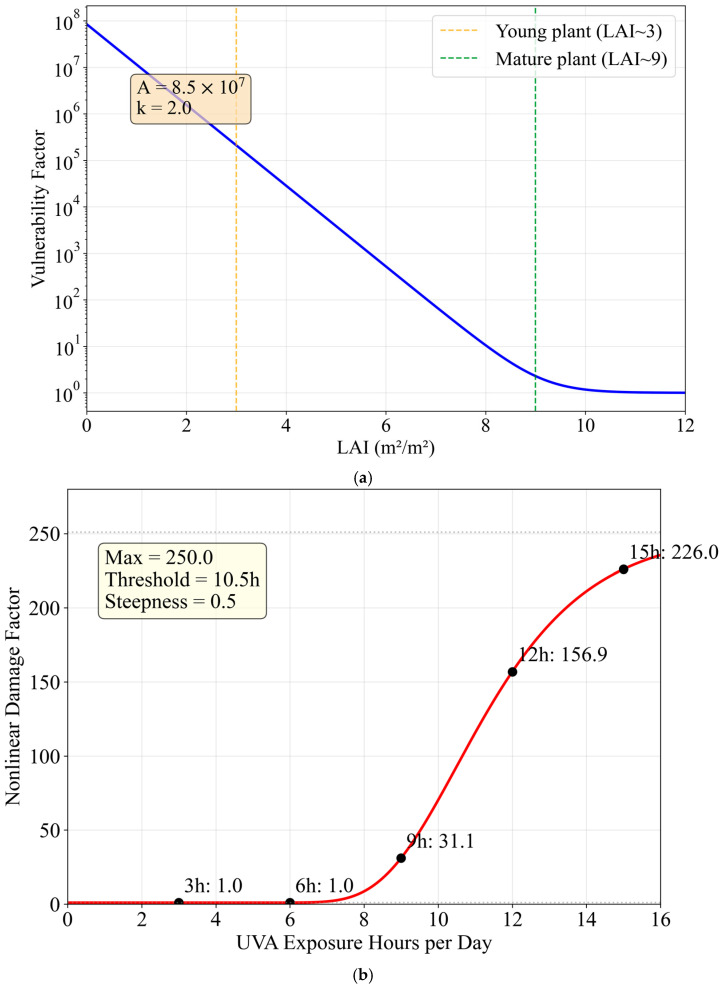

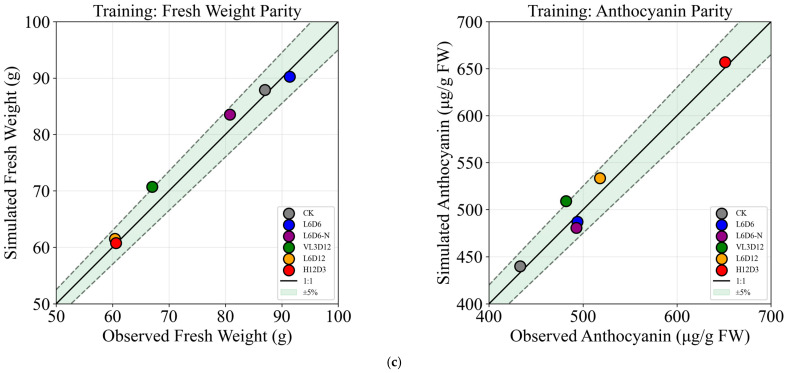
Mechanistic diagnostics. (**a**) LAI vulnerability function v(LAI) = A·exp(−k·LAI) + 1 and the LAI at treatment onset for each recipe. Early-stage treatments (VL3D12, L6D12 starting at Day 23) experience high vulnerability due to low LAI, while late-stage treatments (H12D3 starting at Day 32) benefit from protective canopy development. (**b**) Nonlinear damage amplification as a function of daily irradiation hours modeled by a Gompertz function [25]. The factor remains near 1.0 for ≤6 h day^−1^, rises sharply between 9–12 h day^−1^, and reaches ~227× at 15 h day^−1^ (asymptotic maximum = 251), representing the collapse of protective capacity under prolonged exposure. (**c**) Training set: model prediction vs. observation for fresh weight (**left**) and anthocyanin (**right**). The diagonal is the 1:1 line; shaded bands indicate ±5% error. All training points fall within ±5.6% error, meeting the calibration target.

Model diagnostics indicated that cumulative stress (∫Stress dt) better reflects total stress burden than endpoint stress. Early irradiation (Day 23; VL3D12 and L6D12) incurred high vulnerability at low LAI, causing large cumulative stress despite partial recovery later. The LAI-dependent vulnerability function, nonlinear damage amplification, and training-set calibration accuracy are shown in Figure 10.

The nonlinear damage amplification under prolonged daily exposure follows a Gompertz function [25], with a threshold at approximately 10.5 h day^−1^ (Figure 10b).

**Figure 11:**
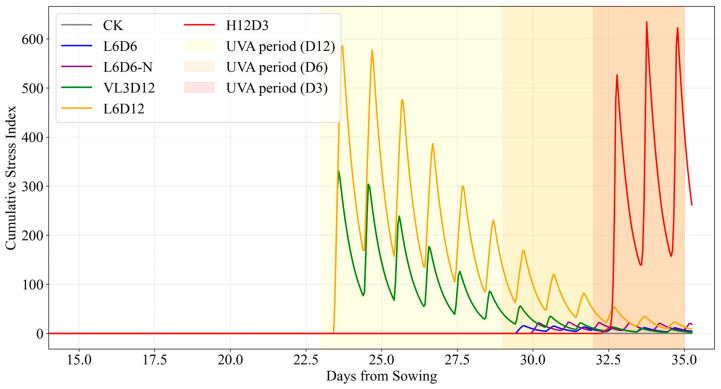
Stress time series for each treatment from Day 14 to Day 35. Vertical dashed lines indicate UV-A onset times. Early-onset treatments (VL3D12, L6D12) show rapid stress accumulation at low LAI, while late-onset treatments (L6D6, H12D3) accumulate stress more gradually due to protective canopy.

Night irradiation increased stress relative to day irradiation, consistent with circadian regulation of ROS homeostasis [26,27,28]. Stress dynamics across treatments are shown in Figure 11.

**Figure 12:**
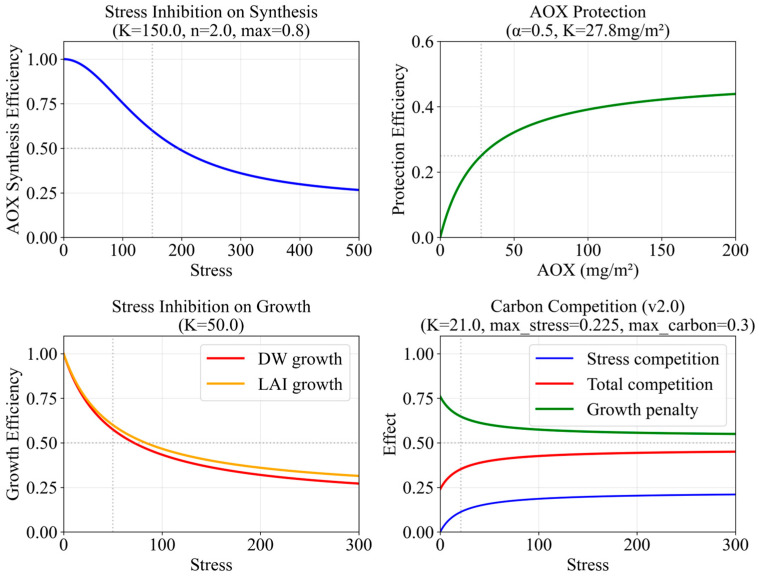
Monotonically decreasing Hill-type inhibition of AOX synthesis efficiency vs. nonlinear damage factor (K = 800, n = 1.5). This smooth inhibition explains the hormesis phenomenon: at extreme doses, stress-induced damage reduces AOX synthesis capacity despite continued UV-A stimulation.

The carbon competition mechanism explains why VL3D12 (low stress) and L6D12 (high stress) both show FW reduction: VL3D12’s stress (Stress = 61) contributes a stress-based competition penalty of ~17%, while L6D12’s higher stress (Stress = 145.5) contributes ~19%. The total carbon competition penalty also includes the AOX carbon demand component. The AOX synthesis efficiency decreases under severe stress following a Hill-type inhibition function [29] (Figure 12).

The system dynamics block diagram illustrating the six-state model structure with feedback loops and carbon competition pathways is provided in Figure S1 (Supplementary Materials). The predicted hormesis response surface across the dose-duration space, demonstrating optimal anthocyanin accumulation at moderate daily hours (8–10 h) with shorter duration (3–5 d), is shown in Figure S2. The carbon competition mechanism visualization, including C_buf dynamics, AOX accumulation trajectories, and growth penalty comparisons across treatments, is provided in Figure S3. A summary of stress diagnostics, carbon competition effects, and dominant mechanisms for each treatment is provided in Table 3.

## 3. Discussion

Experiment 1 confirmed that UV-B is high risk: although it strongly induces pigmentation, it readily causes photodamage and yield loss, whereas UV-A provides a safer window that enhances coloration while preserving biomass, consistent with Weiland et al. [14]. The contrasting responses to these two wavebands reflect fundamental differences in their photobiological mechanisms. UV-B radiation (280–315 nm), with its higher photon energy, can be directly absorbed by DNA, causing pyrimidine dimer formation and triggering signal transduction cascades via the UVR8 photoreceptor pathway [30]. At the physiological level, UV-B exposure induces oxidative stress through the generation of reactive oxygen species, and the balance between pro-oxidant and antioxidant responses determines the extent of acclimation or damage [31], which in severe cases manifests as visible necrotic lesions and tissue dehydration. The resulting increase in leaf dry matter content (LDMC) reflects a disproportionate loss of cellular water relative to structural dry mass—a hallmark of acute photodamage. In our model, this acute LDMC response is captured through the Gompertz-type nonlinear damage amplification factor, which escalates sharply at high daily UV exposure (threshold ≈ 10.5 h day^−1^) and the stress-dependent growth inhibition term. The severe biomass penalty observed under UV-B in all four cultivars is therefore attributable to both direct photodamage and the downstream metabolic costs of damage repair. It should be noted that the UV-B fluorescent lamp used in this study (Sankyo Denki GL-15, peak 302 nm) has a secondary emission component at ~250 nm (UV-C region), which may have contributed to the severity of the observed necrotic lesions. Future studies using narrowband UV-B LEDs (e.g., 310 nm peak) would provide a cleaner comparison; however, this limitation does not affect the main conclusions, as UV-B served solely as a high-stress reference to justify the selection of UV-A for optimization.

In contrast, UV-A radiation (315–400 nm) is perceived primarily through flavin-containing photoreceptors such as cryptochromes and phototropins, eliciting signaling responses that are distinct from the direct macromolecular damage caused by UV-B [15]. At moderate doses, plants can manage the UV-A-induced oxidative load through existing antioxidant defenses while simultaneously exhibiting beneficial morphological acclimation responses. A key observation from Experiment 1 is that UV-A did not reduce fresh weight relative to controls across all four cultivars. This preservation—and in some cases slight enhancement—of biomass under UV-A is attributable to UV-A-induced morphological effects: Chen et al. [17] reported that supplemental UV-A significantly increased leaf area in indoor-cultivated lettuce, and proposed that this enhanced light interception is a key driving force behind the observed biomass increase. This UV-A morphological benefit is represented in our model through the SLAboost and LAIboost terms (Equation (5)), which enhance leaf area development under UV-A irradiation. Lee et al. [18] similarly reported that brief pre-harvest UV-A LED supplementation enhanced nutritional quality (protein and mineral content) of lettuce while maintaining shoot fresh weight, and Lycoskoufis et al. [19] demonstrated that growing lettuce under UV-blocking film followed by targeted pre-harvest UV supplementation increased fresh weight by 30% while restoring phytochemical quality to equivalent levels. These findings collectively support our conclusion that UV-A occupies a distinct physiological niche from UV-B, functioning as a mild eustressor that can enhance secondary metabolism while preserving—or even promoting—vegetative growth.

Cultivar differences further highlight the genotype-specific nature of light-stress responses [20]. Among the four cultivars tested, ‘Lollo Rosso’ exhibited the most favorable combination of robust growth maintenance and strong anthocyanin induction under UV-A, suggesting that its genetic background confers an efficient balance between stress perception and defense activation. This cultivar-specific resilience underscores the necessity of a systematic screening stage before optimization, as a UV recipe effective for one cultivar may be suboptimal or even detrimental to another. Importantly, the findings of Experiment 1—that UV-A is safer than UV-B and that ‘Lollo Rosso’ is a responsive cultivar—directly informed the design of Experiment 2, in which multiple UV-A recipes were tested exclusively on ‘Lollo Rosso’ to explore the dose–response landscape that subsequently motivated and parameterized the mechanistic model.

Experiment 2 demonstrated a classical growth–defense tradeoff [32]. While H12D3 (12 h day^−1^ for 3 days) maximized anthocyanin concentration (651 ppm), it reduced biomass by ~30%, reflecting the severe metabolic cost of high-intensity stress. From a production perspective, total anthocyanin per plant (concentration × fresh weight) is a more relevant metric than concentration alone, because it accounts for the economic value of the entire harvested product. The L6D6 recipe (6 h day^−1^ for 6 days) achieved the best balance: it not only maintained but slightly increased fresh weight (91.4 g vs. 87.0 g in CK), while elevating anthocyanin concentration by 14%. This biomass increase under UV-A treatment is noteworthy and provides direct experimental evidence for the UV-A morphological benefit discussed above. The model attributes this fresh-weight gain to the combined SLAboost and LAIboost effects under low-stress conditions (average Stress = 7.2 for L6D6), where the morphological benefit of enhanced leaf expansion outweighs the modest carbon competition penalty (~7%). In contrast, when the same total UV-A dose (36 h) was delivered as longer daily exposure over fewer days (H12D3: 12 h × 3 d) or extended over a longer period beginning at an early developmental stage (VL3D12: 3 h × 12 d starting Day 23), the stress penalties dominated and fresh weight declined substantially. This dose–timing interaction underscores the importance of optimizing both daily intensity and treatment scheduling, a complexity that motivates the mechanistic modeling approach.

The mechanistic model was built by mapping distinct experimental observations to physiologically motivated terms: ontogeny-dependent vulnerability captured the stronger damage when irradiation began early [33,34]; a Gompertz-type nonlinear factor represented the collapse of protective capacity under prolonged daily exposure [30,31]; and a circadian damage term captured the inferior performance of night irradiation, consistent with clock regulation of ROS homeostasis [26,27,28]. The model’s successful prediction of the 15 h day^−1^ hormesis reversal supports the inclusion of smooth efficiency inhibition under severe stress [23,24].

A key innovation of this model is the explicit representation of carbon competition between growth and antioxidant defense, implementing the Growth-Differentiation Balance Hypothesis [10]. This mechanism provides a physiologically meaningful explanation for the observed growth–defense tradeoff: under UV-A stress, plants allocate carbon from the buffer pool to AOX synthesis, reducing the carbon available for structural growth. The carbon cost of antioxidant synthesis (approximately 1.0 kg C per kg AOX) reflects the substantial metabolic investment in phenylpropanoid biosynthesis, consistent with estimates that up to 20% of photosynthate can be channeled to phenylpropanoids under stress conditions [13]. Recent work on coordinated resource allocation [11] further supports this mechanistic framework, showing that growth–defense tradeoffs arise from fundamental carbon allocation constraints.

**Figure 13:**
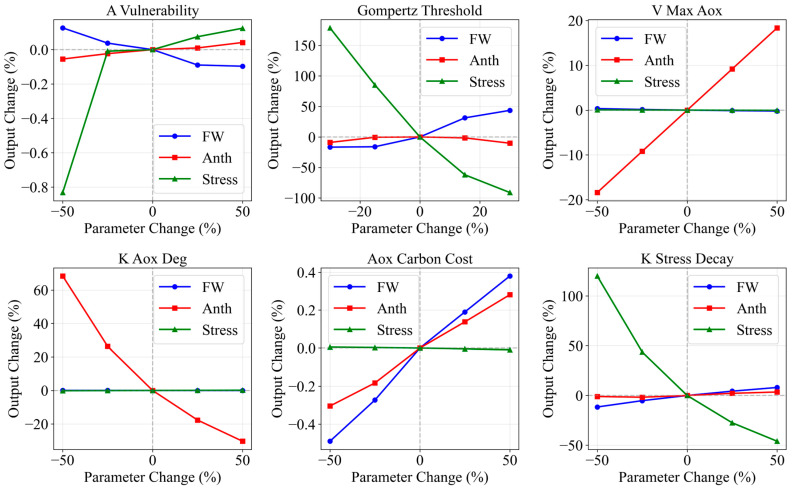
Parameter sensitivity analysis: six-panel plot showing percentage changes in fresh weight (FW), anthocyanin (Anth), and stress level when key model parameters are varied ±25–50% from baseline values under H12D3 treatment conditions. Parameters analyzed include A_vulnerability, gompertz_threshold, V_max_aox, k_aox_deg, aox_carbon_cost, and k_stress_decay.

Sensitivity analysis (Table 4) indicated that parameters governing early-stage vulnerability and the nonlinear damage threshold are most influential for biomass and stress, whereas AOX-specific parameters primarily affect anthocyanin output, suggesting reasonable decoupling between growth and pigment submodules. The carbon competition parameters (stress_competition_K, stress_competition_max) showed moderate sensitivity for both FW and Anth, reflecting their role in coupling growth and defense. The sensitivity plots are provided in Figure 13.

**Figure 14:**
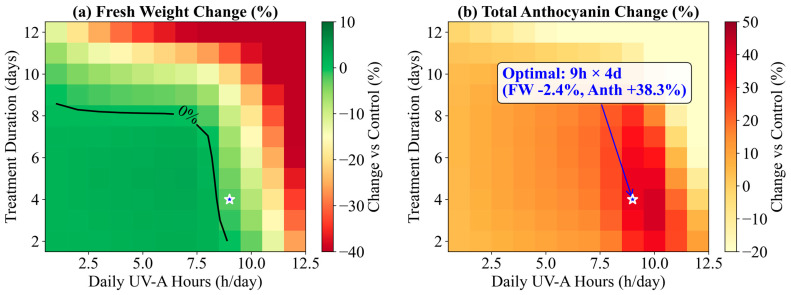
Optimization heatmaps: (**a**) fresh-weight change vs. daily hours and total days; (**b**) total anthocyanin change vs. daily hours and total days. The best strategy (9 h × 4 d, marked with star) maximizes total anthocyanin (+38.3%) while maintaining FW within −5% of control. The white contour in panel (**a**) indicates the −5% FW threshold.

Finally, global search using the calibrated model suggested that 9 h day^−1^ for 4 days is the best theoretical strategy that maximizes total anthocyanin while keeping fresh weight within acceptable limits (≥−5%). This optimum lies below the nonlinear damage threshold region and balances sufficient stress induction with limited growth penalty and carbon competition. The optimization heatmaps are shown in Figure 14, and the top 5 safe strategies are listed in Table 5. Re-running the released simulation code with default numerical tolerances reproduced the same optimum (9 h × 4 d: FW 84.9 g; Anth 613 ppm).

While the present study establishes a proof-of-concept framework, several limitations should be acknowledged to guide future research. First, all experiments were conducted under fixed environmental conditions (temperature 25/18 °C, RH 70/85%, CO_2_ 1200 ppm, EC 1.2 mS cm^−1^). Although this design was necessary to isolate UV-A as the sole experimental variable, it limits the direct applicability of the calibrated parameters to commercial settings where environmental conditions vary. The model architecture—built upon the Sun et al. [22] framework that inherently responds to temperature, CO_2_, and light intensity—supports future extension to multi-factor interactions (UV-A × nutrient composition, UV-A × humidity, UV-A × temperature), provided that corresponding experimental data become available for recalibration.

Second, the baseline PPFD of 130 µmol m^−2^ s^−1^ is at the lower end of commercial lettuce production. This was a practical constraint: accommodating UV-A lamps alongside the LED tubes on the same shelf tier reduced the number of LED tubes per tier. Under higher PPFD, the greater carbon assimilation capacity could shift the growth–defense balance, potentially allowing plants to tolerate higher UV-A doses. Future studies should investigate the interaction between baseline PPFD and UV-A stress response to refine model predictions for higher-intensity production systems.

Third, the core optimization and modeling focused solely on ‘Lollo Rosso.’ Although the two-stage screening–optimization workflow itself is cultivar-agnostic, the calibrated model parameters are cultivar-specific. Extending the framework to other popular red-leaf cultivars (e.g., ‘Red Oakleaf,’ ‘Red Romaine’) through cultivar-specific parameter recalibration is an important direction for future work.

Fourth, the quality assessment focused on fresh weight and anthocyanin concentration as the primary yield and quality indicators. A comprehensive evaluation for commercial applications should include additional nutritional parameters such as vitamin C, nitrate levels, soluble sugars (Brix), and shelf life. Several studies suggest that UV-A supplementation may co-enhance these parameters [18,19], warranting future multi-parameter quality profiling.

Fifth, batch-to-batch variability remains an inherent challenge in plant experimentation. Despite rigorous environmental control, differences in seed lots and germination uniformity can introduce inter-batch variation, as observed between the training and validation control treatments (CK: FW 87.0 vs. 85.2 g; Anth 433 vs. 413 ppm). Multi-batch validation experiments would strengthen confidence in the model’s generalization capability.

Sixth, the interpretation of the hormesis phenomenon (anthocyanin declining at 15 h day^−1^) is based on mechanistic modeling and physiological reasoning rather than direct molecular evidence. Future validation through gene expression analysis (RT-qPCR of PAL, CHS, DFR, ANS), enzymatic activity assays, and direct ROS quantification would substantially strengthen the mechanistic claims and provide independent confirmation of the model’s predictions.

Seventh, the Gompertz-type nonlinear damage threshold (~10.5 h) is an empirically calibrated parameter specific to the current experimental conditions. This threshold would likely shift under different baseline PPFD, physiological stage, or temperature regimes, and recalibration experiments are recommended when conditions differ substantially from the original calibration range.

Finally, the model-predicted optimum (9 h day^−1^ for 4 days) remains a mathematical projection that has not yet been verified through a dedicated physical experiment. Implementing dynamic UV-A recipes in real-world vertical farms also requires real-time sensing of plant stress and growth status. A complementary study from our group on UV-NDVI for real-time crop health monitoring provides a potential non-destructive sensing framework that, when integrated with the present mechanistic model, could enable closed-loop UV-A recipe optimization in commercial production environments.

## 4. Materials and Methods

### 4.1. Cultivation Environment and Equipment

All experiments were conducted in controlled-environment growth chambers at the Department of Bio-Industrial Mechatronics Engineering, National Taiwan University. The hydroponic system consisted of four stacked layers sharing a recirculating nutrient solution to ensure consistent nutrition (Supplementary Figure S5a). Environmental setpoints were as follows: day/night temperature 25/18 ± 1 °C, relative humidity 70/85% (day/night), and CO_2_ concentration 1200 ppm (enriched). Nutrient solution (HuaBao No. 1, Taiwan) was maintained at EC 1.2 mS cm^−1^ and pH 6.5. Baseline lighting was provided by T8 LED tubes (Epistar Corporation, Hsinchu, Taiwan) with a correlated color temperature of 4000 K at a 16 h/8 h photoperiod (06:00–22:00) with PPFD 130 µmol m^−2^ s^−1^ (Table 6). The spectral distribution and spatial irradiance uniformity of the LED tubes are shown in Supplementary Figure S4a,b, respectively.

UV-A irradiation was provided by fluorescent insect-attracting lamps (TOA Lighting Co., Ltd., Taiwan) with a peak emission wavelength of 365 nm, and a canopy-level irradiance of approximately 1100 µW cm^−2.^ UV-B irradiation (used only in the cultivar screening experiment) was provided by UV-B fluorescent lamps (Sankyo Denki Co., Ltd., Kanagawa, Japan; model GL-15) with a peak emission wavelength of 302 nm and a canopy-level irradiance of approximately 1500 µW cm^−2^. For each treatment, UV lamps were mounted alongside the LED tubes on the same shelf tier to ensure uniform irradiance across the plant canopy (Supplementary Figure S5b). Complete spectral emission profiles and spatial irradiance distributions for all light sources are provided in Supplementary Note S1 and Supplementary Figures S6 and S7; a summary of light source specifications is given in Supplementary Table S19.

### 4.2. Experiment 1: Cultivar Screening Under UV-A and UV-B

#### 4.2.1. Plant Materials

The following four red-leaf lettuce cultivars were used: ‘Lollo Rosso’ (LS-006, Known-You Seed, Taiwan), ‘Purple Romaine’ (LE4705, Jiase, Taiwan), ‘Champagne Red Flame’ (Dacheng, Taiwan), and ‘Oakleaf Purple’ (LE4806, Jiase, Taiwan).

#### 4.2.2. Cultivation and UV Treatments

UV treatments started at 32 days after sowing (DAS), and plants were harvested at 35 DAS (n = 5 per treatment for each cultivar). Treatment conditions are summarized in Table 7.

### 4.3. Experiment 2: UV-A Recipe Optimization for the Selected Cultivar

Based on Experiment 1, ‘Lollo Rosso’ was selected for optimization.

#### 4.3.1. Cultivation Procedure

Seeds were soaked for 5 h and sown in sponge cubes. Germination was performed under PPFD 100 µmol m^−2^ s^−1^. Seedlings were transplanted at 14 DAS and harvested at 35 DAS.

#### 4.3.2. Daily UV-A Energy Calculation

To quantify UV-A dose across recipes, daily irradiation energy was calculated as follows:

Edaily = I_UVA (μW cm^−2^) × tdaily (s)

where I_UVA = 1100 µW cm^−2^ = 1.1 × 10^−3^ W cm^−2^ (nominal value for Experiment 2) and tdaily is the daily irradiation time in seconds. Table 8 lists the calculated daily energies for different irradiation durations.

For ecological relevance, winter outdoor UV-A is on the order of 1500–3000 µW cm^−2^ during effective sun hours [15]. Assuming a representative value of 2250 µW cm^−2^ over 6 h (09:30–15:30), the estimated outdoor daily UV-A energy is

Eoutdoor = 2250 (μW cm^−2^) × 21600 (s) × 10^−6^ (W/μW) = 48.6 J cm^−2^

This estimate indicates that the 12 h day^−1^ treatment (47.5 J cm^−2^) is comparable to winter sunlight, supporting the dose range used here [35].

#### 4.3.3. Training-Set Treatments (Optimization Recipes)

UV-A irradiance was fixed at 1100 µW cm^−2^ and different combinations of daily duration and total days were designed (Table 9).

#### 4.3.4. Validation-Set Treatments (3-Day Gradient)

To evaluate generalization, a 3-day gradient experiment was conducted with fixed duration (3 days) and varying daily irradiation time (0–15 h day^−1^), as summarized in Table 10.

### 4.4. Measurements and Data Analysis

The fresh weight (FW) of shoots was measured at harvest. Leaf color parameters (L*, a*, b*) were measured using a colorimeter (NE-4000, Nippon Denshoku, Tokyo, Japan). Anthocyanin content was measured following a modified protocol of Hung et al. [36]: 0.5 g of leaf tissue was homogenized in 5 mL potassium phosphate buffer (pH 6.8), centrifuged at 10,000 rpm for 10 min, and the absorbance of the supernatant was measured at 600 nm. Anthocyanin concentration was calculated using a standard curve prepared with cyanidin-3-glucoside (C3G; Sigma-Aldrich, St. Louis, MO, USA) at concentrations of 0, 50, 100, 200, 400, and 800 mg L^−1^. Final values were expressed as mg kg−1 fresh weight (ppm), calculated as follows: Anth (ppm) = (Cstandard × Vextract)/mtissue, where Cstandard is the concentration from the standard curve (mg L^−1^), Vextract is the extraction volume (L), and mtissue is the tissue mass (kg).

### 4.5. Mechanistic Model of UV-A Effects

A six-state ODE model was developed based on the lettuce growth modeling framework of Sun et al. [22], and extended to represent the dual effects of UV-A as follows: (i) UV-A-induced ROS accumulation leading to stress and growth inhibition, and (ii) UV-A-driven morphological changes that increase leaf area and thereby indirectly enhance PAR interception. A key innovation is the explicit representation of carbon competition between growth and antioxidant defense, implementing the Growth-Differentiation Balance Hypothesis [10].

The model comprises six state variables: structural dry weight (Xd), non-structural carbon buffer (Cbuf), leaf area index (LAI), total antioxidants (AOX), a dimensionless stress state (Stress), and a dimensionless ROS variable (ROS). Anthocyanin concentration is derived as 18% of total AOX, reflecting the proportion of anthocyanins among total antioxidant compounds (flavonoids, ascorbate, etc.).

For reproducibility and to reduce main-text length, the full parameter list, initial conditions, and implementation details are provided in the Supplementary Materials, organized as follows: Table S1 lists all 65 calibrated parameters for the UV-A extension layer grouped by functional category (photosynthesis, UV-A morphological effects, ROS dynamics, stress damage, AOX synthesis/degradation, carbon competition, etc.); Table S2 specifies initial conditions and output conversion formulas; Table S3 details the carbon competition mechanism formulas; Table S4 provides literature support for the carbon competition framework; Table S5 summarizes the Gompertz nonlinear damage factor; Table S6 describes stress dynamics implementation; Table S7 lists all 42 base parameters inherited from the Sun et al. [22] lettuce growth model; Table S8 specifies the environmental conditions used for simulation (including 4 additional environment parameters); Table S9 explains conceptual parameters in the main text equations; Table S10 provides explicit mapping between main text symbols and code variable names; Table S11 specifies the numerical integration method; Table S12 presents the complete Sun model core ODE equations; Table S13 details the stomatal conductance and resistance formulas; Table S14 describes the energy balance approach for leaf temperature; Table S15 provides the complete AOX synthesis formula with all efficiency modulation factors; Table S16 describes the AOX consumption formula with ROS-dependent Hill function; Table S17 details the LDMC dynamics formula; and Table S18 explains UV-A morphological effects with stress suppression.

#### 4.5.1. State Variables

The six state variables are as follows: Xd (structural dry weight, kg m^−2^), Cbuf (non-structural carbon buffer, kg m^−2^), LAI (m^2^ m^−2^), AOX (total antioxidants, kg m^−2^), Stress (dimensionless), and ROS (dimensionless). Anthocyanin is calculated as Anth = AOX × 0.18.

#### 4.5.2. Photosynthesis and Energy Conversion

The baseline growth model follows Sun et al. [22] and retains their energy balance and stomatal conductance formulation. Canopy photosynthesis is based on the Farquhar biochemical model [37] and integrates canopy layers using three-point Gaussian quadrature. Rubisco-limited and RuBP-regeneration-limited assimilation rates are as follows:

Ac = Vc,max (Ci − Γ*)/(Ci + Kc (1 + O/Ko)),(1)

Aj = J (Ci − Γ*)/(4 Ci + 8 Γ*),(2)

where J is the electron transport rate dependent on irradiance and temperature.

To harmonize units for artificial lighting, PPFD was converted to PAR power using a spectrum-weighted coefficient kappa based on Table 6 as follows:

κ = 0.30 × 0.181 + 0.45 × 0.217 + 0.25 × 0.266 = 0.219 J μmol^−1^

Thus, PPFD = 130 μmol m^−2^ s^−1^ corresponds to PAR ≈ 28.5 W m^−2^. Because the Sun model assumes PAR = σPAR × I with σPAR = 0.5, we used an equivalent shortwave irradiance ILED = 57 W m^−2^ for the baseline light.

UV-A at 365 nm does not directly contribute to photosynthesis and is introduced as a driver of ROS production and as a trigger for morphological effects. The measured UV-A intensity was I_UVA = 1100 µW cm^−2^.

#### 4.5.3. Core ODE System

Carbon buffer dynamics with carbon competition:

dCbuf/dt = (calpha · AC · hbuf − Rd) − (RGRmax · Xd/cbeta) − SAOX · Ccost,(3)

where AC is canopy photosynthesis rate, hbuf is the carbon buffer feedback term (see Table S12), Rd is dark respiration, RGRmax is the maximum relative growth rate (from the Sun model), cα = 0.54 and cβ = 0.8 are allocation coefficients from the Sun model, SAOX is the AOX synthesis rate, and Ccost = 1.0 kg C per kg AOX represents the carbon cost of antioxidant synthesis. The second term represents structural carbon consumption for growth (consistent with Sun Equation (S7) in Table S12). This formulation explicitly implements the carbon competition between growth and defense [10,11].

Structural growth dynamics with carbon competition penalty:

dXd/dt = [Cbuf/(Cbuf + Kcarbon)] × RGRmax × Xd × (1 − Stressinhib) × (1 − CCpenalty),(4)

The carbon competition penalty is calculated as follows:

CC_penalty_ = (AOX_carbon_effect_ × 0.30) + (0.225 × Stress/(21 + Stress))

where AOX_carbon_effect_ is a Hill-type saturation function of stress-induced AOX synthesis carbon demand.

AOXcarbon_effect = DAOX/(Kcc + DAOX)

DAOX = SAOX,stress × Ccost

Here, SAOX,stress is the stress-induced component of AOX synthesis rate (kg m^−2^ s^−1^), Ccost = 1.0 kg C per kg AOX is the carbon cost of synthesis, and Kcc = 1 × 10^−8^ kg m^−2^ s^−1^ is the half-saturation constant. This Hill function ensures smooth transitions and bounded effects (0 ≤ AOXcarbon_effect ≤ 1). The first term (×0.30) represents the direct carbon diversion to AOX synthesis, while the second term represents the indirect effect of cumulative stress on resource allocation [11].

LAI dynamics:

dLAI/dt = (dXd/dt) · SLA · (1 − σr) · (1 + LAIboost),(5)

SLAboost = αSLA · I_UVA/(KSLA + I_UVA),

LAIboost = αLAI · I_UVA/(KLAI + I_UVA)

where SLA is the environment-dependent specific leaf area from the Sun model, σr is the shoot:root ratio, and the UV-A morphological boosts (SLAboost applied to dXd/dt, LAIboost applied to dLAI/dt) enhance leaf expansion under UV-A. Under high stress, these morphological benefits are suppressed (see Table S18 for complete formulation). The Sun model internally handles leaf turnover through the shoot:root allocation dynamics.

ROS dynamics:

dROS/dt = kprod × I_UVA − kclear × ROS,(6)

Stress dynamics:

dStress/dt = (Damage + Circadian_Damage) − kdecay · Stress,(7)

Damage = (Damagevuln + Damagenonlin) · (1 − AOXprotection)

Damagevuln = stress_coeff · ROS · vulnerability(LAI)

Damagenonlin = knonlin · ROS · Nonlinear_Factor

vulnerability(LAI) = Avuln · exp(−kvuln · LAI) + 1

Nonlinear_Factor = 1 + Fmax · exp(−exp(−ksteep · (Hours_today − Hthreshold)))

AOXprotection = αprot · AOX / (Kprot + AOX)

Circadian_Damage = kcirc · I_UVA · Hours_in_Darkn_circ

(night-only, not protected by AOX)

where Hours_today is the cumulative UV-A exposure within the current day (0 to daily_hours), stress_coeff = 1.6 × 10^−7^, knonlin = 5.0 × 10^−6^, Avuln = 8.5 × 10^7^, kvuln = 2.0, Fmax = 250, Hthreshold = 10.5 h, ksteep = 0.5 h^−1^, αprot = 0.5, Kprot = 2.78 × 10^−5^ kg m^−2^, and kdecay = 2.14 × 10^−5^ s^−1^ (half-life ~9 h). The two damage components (vulnerability-based and nonlinear amplification) are additive with separate coefficients, reflecting distinct physiological mechanisms.

(8) Anthocyanin dynamics:

dAOX/dt = Synthesis − Degradation − Consumption,(8)

AOX synthesis includes baseline, stress-induced and UV-induced components [38,39]. Anthocyanins constitute approximately 18% of total antioxidants, so Anth = AOX × 0.18. AOX consumption represents antioxidant usage for ROS scavenging under high oxidative pressure [40,41,42]. Additional smooth inhibition terms represent reduced synthetic efficiency under severe dehydration [24,43] and reduced carbon supply under low LAI [44,45].

#### 4.5.4. Carbon Competition Mechanism

The carbon competition mechanism is a key innovation of this model, grounded in the Growth-Differentiation Balance Hypothesis [10]. Under UV-A stress, plants face a tradeoff: allocate carbon to growth (Xd) or to defense (AOX synthesis). This is implemented through two pathways:

1. Direct carbon consumption: AOX synthesis consumes carbon from Cbuf at a rate proportional to synthesis rate × carbon cost (1.0 kg C per kg AOX). The carbon cost reflects the metabolic investment in phenylpropanoid biosynthesis, which can consume up to 20% of photosynthate [13].

2. Growth penalty: High stress and AOX synthesis rates reduce growth efficiency through the CCpenalty term, reflecting resource allocation to defense at the expense of growth [12].

The carbon consumption for AOX synthesis is subject to a maximum rate constraint:

AOX_carbon_consumption = min(S_AOX × C_cost, C_buf × 0.10/τ)

where τ = 300 s is the carbon buffer turnover time constant, ensuring dimensional consistency (kg C s^−1^) with the continuous ODE formulation.

### 4.6. Statistical Analysis

Experimental data are presented as mean ± SD (n = 5). One-way ANOVA followed by Tukey’s HSD test was performed in IBM SPSS Statistics (version 29; IBM Corp., Armonk, NY, USA) (*p* < 0.05).

## 5. Conclusions

We developed a two-stage framework integrating experiments and mechanistic modeling to enhance anthocyanin in red-leaf lettuce without yield loss. Experiments showed that UV-A is safer than UV-B and identified ‘Lollo Rosso’ as a highly responsive cultivar. Among tested recipes, L6D6 (6 h day^−1^ for 6 days) maintained fresh weight while increasing anthocyanin concentration and total anthocyanin per plant.

The six-state mechanistic ODE model, featuring explicit carbon competition between growth and antioxidant defense based on the Growth-Differentiation Balance Hypothesis [10], reproduced training data with <5% error in 10 of 12 metrics and predicted an independent validation dataset with <10% error in 11 of 12 metrics. The model captures key nonlinear features including ontogeny-dependent vulnerability, nonlinear damage amplification, circadian disruption, carbon competition, and hormesis at extreme doses.

Global optimization predicted that 9 h day^−1^ for 4 days is the theoretical optimum, increasing total anthocyanin by ~38% (relative to control) with minimal yield reduction (−2.4%), and warrants future experimental verification.

Overall, the workflow of “experimental observation → mechanistic hypothesis → mathematical formulation → data-driven calibration → model-based optimization” offers a general paradigm for resolving yield–quality tradeoffs in controlled-environment agriculture. The carbon competition framework provides a mechanistic basis for understanding and optimizing the growth–defense balance in crops. Key priorities for future work include experimental verification of the model-predicted optimum, multi-factor interaction studies across broader environmental conditions, extension to additional cultivars, comprehensive quality profiling (vitamin C, nitrates, Brix), and molecular validation of the hormesis mechanism through enzymatic and gene expression assays.

## Figures and Tables

**Table 1:** Training-set calibration results.

Treatment	FW Obs (g)	FW Pred (g)	FW Error	Anth Obs (ppm)	Anth Pred (ppm)	Anth Error	Avg Stress
CK	87.0	87.9	+1.0%	433	440	+1.6%	0.0
L6D6	91.4	90.2	−1.3%	494	487	−1.4%	7.2
L6D6-N	80.8	83.5	+3.4%	493	481	−2.5%	10.5
VL3D12	67.0	70.7	+5.6%	482	509	+5.6%	61.0
L6D12	60.4	61.5	+1.8%	518	533	+2.9%	145.5
H12D3	60.6	60.9	+0.6%	651	655	+0.6%	262.0

**Table 2:** Validation-set prediction results.

Treatment	Hours	FW Obs (g)	FW Pred (g)	FW Error	Anth Obs (ppm)	Anth Pred (ppm)	Anth Error
CK_val	0	85.2	87.9	+3.2%	413	440	+6.5%
VL3D3	3	89.0	88.5	−0.7%	437	460	+5.4%
L6D3	6	92.2	89.1	−3.4%	468	480	+2.5%
M9D3	9	83.8	85.9	+2.5%	539	596	+10.6%
H12D3_val	12	62.2	60.9	−2.0%	657	655	−0.3%
VH15D3	15	51.3	51.2	+0.0%	578	605	+4.8%

**Table 3:** Stress diagnostics, carbon competition effects, and dominant mechanisms.

Treatment	Pattern	Total Hours	Avg Stress	Carbon Competition	Dominant Mechanisms
CK	none	0	0.0	0%	control
L6D6	6 h × 6 d	36	7.2	~7%	low daytime dose, minimal penalty
L6D6-N	6 h × 6 d (night)	36	10.5	~10%	circadian disruption
VL3D12	3 h × 12 d	36	61.0	~17%	early-stage vulnerability, carbon competition
L6D12	6 h × 12 d	72	145.5	~19%	cumulative stress + carbon competition
H12D3	12 h × 3 d	36	262.0	~20%	nonlinear damage + acute LDMC

**Table 4:** Sensitivity analysis of key parameters.

Parameter	Category	S_FW	S_Stress	S_Anth	Sensitivity
A_vulnerability	LAI vulnerability	−1.28	+13.2	+1.60	Very high
gompertz_threshold	Nonlinear threshold	−0.85	+8.5	+0.92	High
V_max_aox	Stress-induced synthesis	~0	~0	+0.85	High
k_aox_deg	AOX degradation	~0	~0	−0.95	High
base_aox_rate_light	Baseline synthesis (light)	~0	~0	+0.75	High
stress_competition_K	Carbon competition	−0.35	+1.2	−0.25	Medium
K_nonlin_aox	Nonlinear inhibition	~0	~0	+0.30	Medium
k_aox_consumption	AOX consumption	~0	~0	−0.60	Medium
k_stress_decay	Stress decay	+0.45	−2.8	−0.35	Medium

**Table 5:** Top 5 safe UV-A strategies predicted by global search (FW ≥ −5% vs. control). Percent changes are relative to the experimental control at harvest (CK: FW = 87.0 g, Anth = 433 ppm).

Rank	Hours per Day	Total Days	Start Day	FW (g)	FW Change	Anth (ppm)	Anth Change	Total Anth Change
1	9	4	Day 31	84.9	−2.4%	613	+41.6%	+38.3%
2	9	5	Day 30	84.0	−3.5%	620	+43.1%	+38.1%
3	9	6	Day 29	82.8	−4.9%	620	+43.3%	+36.3%
4	10	2	Day 33	84.2	−3.2%	610	+40.9%	+36.3%
5	9	3	Day 32	85.9	−1.3%	596	+37.7%	+36.0%

**Table 6:** Spectral composition of the primary LED lighting used for cultivation.

LED Type	Red (%)	Green (%)	Blue (%)	PPFD (μmol m^−2^ s^−1^)
LED 4000K	30	45	25	130

**Table 7:** Treatment conditions for the cultivar screening experiment (Experiment 1).

Code	Description	Lamp Type	Peak Wavelength (nm)	Irradiance (μW cm^−2^)	Hours per Day (h)	Duration (days)
UV-A	UV-A irradiation	Fluorescent insect-attracting lamp (TOA Lighting)	365	1104	4	3
UV-B	UV-B irradiation	UV-B fluorescent lamp (Sankyo Denki, GL-15)	302	1510	4	3
CK	Control (no UV)	-	-	-	-	-

**Table 8:** Daily UV-A energy calculation for I_UVA = 1100 µW cm^−2^.

Daily Duration (h)	Seconds per Day (s)	Daily Energy (J cm^−2^)
3	10,800	11.9
6	21,600	23.8
9	32,400	35.6
12	43,200	47.5
15	54,000	59.4

**Table 9:** Training-set UV-A recipes for ‘Lollo Rosso’ (Experiment 2).

Code	Description	I_UVA (µW cm^−2^)	Hours per Day (h)	Total Days (d)	Start Day
CK	Control (no UV-A)	0	0	0	-
UV-A-L6D6	Low daily dose	1100	6	6	Day 29
UV-A-L6D6-N	Night irradiation	1100	6 (night)	6	Day 29
UV-A-H12D3	High daily dose	1100	12	3	Day 32
UV-A-VL3D12	Very low daily dose (long)	1100	3	12	Day 23
UV-A-L6D12	Low daily dose (long)	1100	6	12	Day 23

**Table 10:** Validation-set UV-A recipes (3-day gradient).

Code	Description	I_UVA (µW cm^−2^)	Hours per Day (h)	Total Days (d)	Start Day	Daily Energy (J cm^−2^)
CK	Control	0	0	0	-	0
VL3D3	Very low daily dose	1100	3	3	Day 32	11.9
L6D3	Low daily dose	1100	6	3	Day 32	23.8
M9D3	Medium daily dose	1100	9	3	Day 32	35.6
H12D3	High daily dose	1100	12	3	Day 32	47.5
VH15D3	Very high daily dose	1100	15	3	Day 32	59.4

## Data Availability

The data supporting the findings of this study are available within the article and its Supplementary Materials. Complete model parameters are provided in Tables S1–S18, system dynamics diagrams in Figures S1–S3, and detailed mathematical formulations in the Supplementary Materials. The Python (version 3.13.12; Python Software Foundation) implementation of the six-state ODE model, using SciPy (version 1.17.1) for numerical integration and NumPy (version 2.4.2) for computation, including all scripts for parameter optimization and data visualization, is publicly available at https://github.com/GrayWei444/uva-simulation (accessed on 29 January 2025). Raw experimental data are available from the corresponding authors upon reasonable request.

## Supplementary Materials

The following supporting information can be downloaded at: https://www.mdpi.com/article/10.3390/plants15050838/s1. Table S1. Complete parameter set for the mechanistic ODE model (Carbon Competition + AOX Framework), listing all 65 calibrated parameters grouped by functional category. Table S2. Initial conditions and output conversion for simulation. Table S3. Carbon competition mechanism parameters and formulas, including Equations (S1)–(S3). Table S4. Literature support for the carbon competition mechanism. Table S5. Nonlinear damage factor (Gompertz function), Equation (S4). Table S6. Stress dynamics implementation detail, Equation (S5). Table S7. Sun model base parameters (from Sun et al. [22]). Table S8. Environment conditions for simulation. Table S9. Conceptual parameters in main text equations. Table S10. Main text symbol to code variable correspondence. Table S11. Numerical integration method. Table S12. Sun model core ODE equations, Equations (S6)–(S15). Table S13. Stomatal conductance and resistance formulas, Equations (S16)–(S20). Table S14. Energy balance and leaf temperature. Table S15. Complete AOX synthesis formula, Equation (S21). Table S16. AOX consumption formula, Equation (S22). Table S17. LDMC dynamics formula, Equation (S23). Table S18. UV-A morphological effects with stress suppression, Equations (S24)–(S25). Table S19. Summary of light source specifications. Note S1. Experimental light sources and cultivation setup. Figure S1. System dynamics block diagram illustrating the six-state ODE model structure with carbon competition mechanism. Figure S2. Hormesis response surface showing predicted anthocyanin concentration as a function of daily UV-A hours and treatment days. Figure S3. Carbon competition mechanism visualization under different UV-A treatments: (a) carbon buffer dynamics, (b) AOX accumulation, (c) carbon competition penalty, (d) growth penalty comparison. Figure S4. Primary LED light source characterization: (a) spectral distribution, (b) spatial irradiance distribution. Figure S5. Cultivation shelf and lamp configuration: (a) multi-tier shelf system, (b) lamp positioning. Figure S6. UV lamp spectral distributions: (a) UV-A lamp (365 nm peak), (b) UV-B lamp (302 nm peak). Figure S7. UV lamp spatial irradiance distributions: (a) UV-A, (b) UV-B.
