# Supplementary material for: A Two-Stage Screening-to-Optimization Approach with Mechanistic Model Analysis: Enhancing Anthocyanin in Lettuce Without Yield Loss
Source: Plants (Basel). 2026 Mar 9;15(5):838. doi: 10.3390/plants15050838 (PMC12986601; doi:10.3390/plants15050838)
Supplement: Supplementary file 1 [file plants-15-00838-s001.zip › plants-4162170 Supplementary Materials.pdf]

Supplementary Materials

Mechanistic Model Parameters and Implementation Details

UV-A Extension Layer · Carbon Competition + AOX Framework

Notation Convention

Symbol	Meaning
Lowercase k	Rate constants or coefficients
Uppercase K	Half-saturation constants (Michaelis-Menten)
α, β (Greek)	Scaling factors
Code variable (e.g. uva_lai_boost)	Corresponds to math symbol (e.g. α_LAI) in Equation 5

Table S1 Complete Parameter Set (65 Parameters)

UV-A extension layer parameters grouped by functional category. Additional 4 environment parameters (PPFD, I_base, I_UVA, plant_density) are specified in Table S8.

Notation: Lowercase k = rate constants; Uppercase K = half-saturation constants; Greek α, β = scaling factors.

Cat.	Parameter	Description	Value	Unit
A	c_alpha	Photosynthetic efficiency scaling factor	0.54	—
B	uva_sla_enhancement (α_SLA)	Maximum UV-A enhancement on SLA	5.0	—
B	K_uva_sla (K_SLA)	Half-saturation UV-A intensity for SLA enhancement	7.5	$W\ m^{-2}$
B	uva_lai_boost (α_LAI)	Maximum UV-A boost on LAI growth	1.70	—
B	K_uva_lai (K_LAI)	Half-saturation UV-A intensity for LAI boost	7.5	$W\ m^{-2}$
C	k_ros_production	ROS production coefficient	0.010	ROS $(W\ m^{-2}\ s)^{-1}$
C	k_ros_clearance	ROS clearance coefficient	5×10^{-4}	s^{-1}
D	stress_damage_coeff	Baseline damage coefficient	1.6×10^{-7}	Stress $(ROS\ s)^{-1}$
D	A_vulnerability	Amplitude of LAI-dependent vulnerability	8.5×10^{7}	—
D	k_vulnerability	Decay of vulnerability with LAI	2.0	—

Cat.	Parameter	Description	Value	Unit
D	gompertz_max_factor	Maximum damage amplification (upper bound)	250.0	—
D	gompertz_threshold	Gompertz inflection (collapse time)	10.5	h
D	gompertz_steepness	Gompertz steepness	0.5	h^{-1}
D	alpha_aox_protection	Maximum AOX protection efficiency	0.5	—
D	K_aox_protection	Half-saturation for AOX protection	2.78×10^{-5}	kg m^{-2}
D	k_circadian	Circadian damage coefficient (night irradiation)	3.0×10^{-6}	—
D	n_circadian	Exponent for circadian damage	2.0	—
D	k_nonlinear_stress	Nonlinear stress coefficient	5.0×10^{-6}	—
E	k_stress_decay	Stress decay rate (half-life ~9 h)	2.14×10^{-5}	s^{-1}
F	stress_photosynthesis_inhibition	Maximum photosynthesis inhibition by stress	0.85	—
F	stress_lai_inhibition	Maximum LAI-growth inhibition by stress	0.80	—
F	K_stress	Half-saturation stress level for growth inhibition	50.0	—
G	base_aox_rate_light	Baseline AOX synthesis rate (light)	3.53×10^{-9}	kg (m^2 LAI s)$^{-1}$
G	base_aox_rate_dark	Baseline AOX synthesis rate (dark)	1.77×10^{-9}	kg (m^2 LAI s)$^{-1}$
G	V_max_aox	Maximum stress-induced AOX synthesis rate	1.45×10^{-8}	kg (m^2 LAI s)$^{-1}$
G	K_stress_aox	Half-saturation for stress-induced synthesis	100.0	—
G	k_aox_deg	AOX degradation rate	3.02×10^{-6}	s^{-1}
G	k_aox_consumption	AOX consumption coefficient (ROS scavenging)	1.8×10^{-7}	s^{-1}
G	k_uv_aox	UV-induced AOX synthesis coefficient	7.78×10^{-11}	kg m^{-2} s^{-1}
G	K_uv_hours	Half-saturation for UV hours effect	30.0	h
H	water_aox_threshold	LDMC threshold triggering water inhibition (DW/FW)	0.055	—
H	water_aox_K	Half-saturation for water inhibition	0.020	—
H	water_aox_max_inhib	Maximum water inhibition on synthesis	0.50	—
H	water_n	Hill coefficient for water inhibition	2.0	—
I	K_stress_inhib	Half-saturation for stress inhibition	150.0	—

Cat.	Parameter	Description	Value	Unit
		on synthesis		
I	n_stress_inhib	Hill exponent for stress inhibition	2.0	—
I	max_stress_inhib	Maximum stress inhibition on synthesis	0.80	—
J	K_nonlin_aox	Half-effect constant for nonlinear efficiency	800.0	—
J	n_nonlin_aox	Hill exponent for nonlinear efficiency	1.5	—
K	K_adapt_days	Half-saturation days for adaptation effect	4.0	days
L	dw_fw_ratio_base	Baseline DW/FW ratio (healthy plants)	0.05	—
L	ldmc_stress_sensitivity	Stress sensitivity of LDMC	0.45	—
L	K_ldmc	Half-saturation for LDMC effect	1400.0	—
L	dw_fw_ratio_max	Maximum DW/FW ratio (severe stress)	0.080	—
L	acute_center	Softplus center for acute LDMC	50.0	—
L	acute_scale	Softplus scale for acute LDMC	10.0	—
L	acute_k	Acute LDMC coefficient	9.0	—
L	acute_K	Acute LDMC half-saturation	120.0	—
L	acute_n	Acute LDMC Hill coefficient	2.0	—
M	LAI_healthy	Reference LAI for healthy plant	9.0	$m^2\ m^{-2}$
M	n_LAI_eff	Hill coefficient for LAI efficiency	2.0	—
M	night_stress_efficiency	Night irradiation stress efficiency	0.4	—
N	K_ros_consumption	ROS half-saturation for AOX consumption	500.0	—
N	n_ros_consumption	Hill coefficient for ROS consumption	2.0	—
N	cons_amp_center	Softplus center for consumption amplification	200.0	—
N	cons_amp_scale	Softplus scale for consumption amplification	15.0	—
N	cons_amp_k	Amplification coefficient	12.0	—
N	cons_amp_K	Amplification half-saturation	20.0	—
O	aox_carbon_cost	Carbon cost per AOX synthesized	1.0	kg C / kg AOX
O	carbon_competition_K	Half-saturation for AOX carbon effect	1×10^{-8}	$kg\ m^{-2}\ s^{-1}$
O	stress_competition_K	Half-saturation for stress competition	21.0	—

Cat.	Parameter	Description	Value	Unit
O	stress_competition_max	Maximum stress-based competition	0.225	—
O	carbon_competition_max	Maximum AOX-based competition	0.30	—
O	max_cbuf_consumption	Max fraction of C_buf consumed per timestep τ (τ=300s)	0.10	—
P	anthocyanin_fraction	Fraction of AOX that is anthocyanin	0.18	—

Table S2 Initial Conditions & Output Conversion

Simulation spans 14–35 DAS. FW_init = 10 g plant^{-1}, LDMC_base = 0.05 (DW/FW).

Initial Conditions

State Variable	Formula	Value
X_d(0)	FW_init × LDMC_base × plant_density / 1000 = 10 × 0.05 × 36 / 1000	0.018 kg m^{-2}
C_buf(0)	0.1 × X_d(0)	0.0018 kg m^{-2}
LAI(0)	X_d(0) × SLA_transplant = 0.018 × 111	≈ 2.0 m^2 m^{-2}
AOX(0)	—	1.0×10^{-5} kg m^{-2} (~5 ppm anthocyanin)
Stress(0)	—	0
ROS(0)	—	0

Note: SLA_transplant = 111 m^2 kg^{-1} (≈2.3× SLA_ref); reflects thinner, more expanded leaves of young seedlings under controlled-environment conditions. Consistent with reported ontogenetic SLA decline in lettuce.

Output Conversion

Output	Formula
FW (g plant^{-1})	[X_d* / (plant_density × LDMC)] × 1000
Anth_ppm (mg kg^{-1})	(AOX* × 0.18 / FW_total) × 10^6 where FW_total = FW × plant_density / 1000 (kg m^{-2})

Table S3 Carbon Competition Mechanism

Implements the Growth-Differentiation Balance Hypothesis (Herms & Mattson, 1992).

Equation S1 — Carbon Competition Penalty

stress_carbon_effect = 0.225 × Stress / (21 + Stress)

aox_carbon_effect = stress_aox_carbon_demand / (carbon_competition_K + stress_aox_carbon_demand)

CC_penalty = aox_carbon_effect × 0.30 + stress_carbon_effect

growth_penalty = 1.0 − CC_penalty

dX_d/dt = dX_d/dt × growth_penalty (S1)

Equation S2 — Real C_buf Consumption

max_consumption_rate = C_buf × 0.10 / 300 (kg C s^{-1})

aox_carbon_consumption = min(aox_synthesis_rate × aox_carbon_cost, max_consumption_rate)

dC_buf/dt = dC_buf/dt − aox_carbon_consumption (S2)

Equation S3 — AOX Synthesis Penalty (Feedback Inhibition)

aox_synthesis_penalty = 1.0 − 0.20 × CC_penalty

S_AOX_final = S_AOX_total × aox_synthesis_penalty (S3)

> *Note: τ = 300 s is the carbon buffer turnover time constant, ensuring dimensional consistency. The penalty applies to total AOX synthesis as all biosynthesis pathways share the same carbon pool. This reflects reduced synthetic capacity when carbon is limiting, consistent with metabolic regulation under resource constraints.*

Table S4 Literature Support for Carbon Competition

Mechanism	Reference	DOI
Growth-Differentiation Balance Hypothesis	Herms & Mattson (1992)	10.1086/417659
Coordinated resource allocation	Monson et al. (2022)	10.1111/nph.17773
Phenylpropanoid carbon flux (~20% photosynthate)	Vogt (2010)	10.1093/mp/ssp106
Metabolic costs of secondary metabolites	Gershenzon (1994)	10.1007/BF02059810

Table S5 Nonlinear Damage Factor (Gompertz Function)

Equation S4

factor = 1 + 250 × exp(−exp(−0.5 × (hours − 10.5)))

Daily Hours (h)	Gompertz Factor
3	1.0
6	1.0
9	31.1
12	156.9
15	226.7

> *Note: The factor is calculated using hours_today (current exposure progress within the day) for progressive damage accumulation. The table shows final daily values for reference.*

Table S6 Stress Dynamics Implementation

Equation S5 — Stress Accumulation

vuln_damage = stress_damage_coeff × ROS × vulnerability(LAI)

nonlin_damage = k_nonlinear_stress × ROS × Nonlinear_Factor

base_damage = vuln_damage + nonlin_damage

protected_damage = base_damage × (1 − aox_protection)
total_damage = protected_damage + circadian_damage
dStress/dt = total_damage − k_stress_decay × Stress (S5)

Component	Description
vuln_damage	LAI-dependent vulnerability effect
nonlin_damage	Gompertz-modulated nonlinear damage amplification
base_damage	Combined damage before AOX protection
protected_damage	Base damage reduced by AOX protection (applies to both vuln. and nonlinear components)
circadian_damage	Additional damage for night irradiation — not protected by AOX (distinct physiological pathway)

Table S7 Sun Model Base Parameters (Sun et al., 2025)

Parameters inherited from the calibrated lettuce growth framework of Sun et al. (2025).

Category	Parameter	Description	Value	Unit
Physical	M_CO2	Molar mass of CO_2	44×10^{-3}	kg mol^{-1}
Physical	Rg	Universal gas constant	8.314	J mol^{-1} K^{-1}
Physical	T0_K	Reference temperature (0°C)	273.15	K
Physical	T25_K	Reference temperature (25°C)	298.15	K
C allocation	c_alpha	Photosynthetic efficiency (internal)	0.68	—
C allocation	c_beta	Growth respiration coefficient	0.8	—
C allocation	sigma_buf	Max C_buf as fraction of X_d	0.2	—
Growth rate	RGR_max_20	Max relative growth rate at 20°C	1.54×10^{-6}	s^{-1}
Growth rate	Q10_gr	Q10 for growth	1.6	—
Growth rate	T_c_RGR	Critical temperature for RGR	25.0	°C
Respiration	c_Rd_25_sh	Shoot dark respiration at 25°C	3.47×10^{-7}	kg m^{-2} s^{-1}
Respiration	c_Rd_25_r	Root dark respiration at 25°C	1.16×10^{-7}	kg m^{-2} s^{-1}
Respiration	Q10_Rd	Q10 for respiration	2.0	—
Light	cr_I	Canopy reflection coeff. (total)	0.22	—
Light	cr_PAR	Canopy reflection coeff. (PAR)	0.07	—
Light	kI	Extinction coefficient (total)	0.48	—
Light	kPAR	Extinction coefficient (PAR)	0.9	—
Light	sigma_PAR	PAR fraction of shortwave	0.5	—
SLA	SLA_ref	Reference specific leaf area	47.93	m^2 kg^{-1}
SLA	Ia_L_ref	Reference leaf irradiance	50.3	W m^{-2}

Category	Parameter	Description	Value	Unit
SLA	Xh_ref	Reference relative humidity	0.75	—
SLA	beta_I	SLA irradiance sensitivity	-4.74×10^{-3}	—
SLA	beta_Xh	SLA humidity sensitivity	0.912	—
Shoot:root	c_sigma_r_1	Shoot ratio coefficient 1	−0.026	—
Shoot:root	c_sigma_r_2	Shoot ratio coefficient 2	−0.076	—
Photosyn.	epsilon_0	Quantum efficiency coefficient	17×10^{-9}	kg J^{-1}
Photosyn.	Gamma_T20	CO_2 compensation point at 20°C	40.0	ppm
Photosyn.	Q10_Gamma	Q10 for compensation point	2.0	—
Photosyn.	Jmax_25	Max electron transport at 25°C	210.15	µmol m^{-2} s^{-1}
Photosyn.	EJ	Activation energy for Jmax	3.7×10^{4}	J mol^{-1}
Photosyn.	cH	Enthalpy for Jmax temp. response	2.2×10^{5}	J mol^{-1}
Photosyn.	cS	Entropy for Jmax temp. response	710.0	J mol^{-1} K^{-1}
Stomatal	c_zeta	Stomatal conductance coefficient	1.6	—
Stomatal	r_H2O_min	Minimum stomatal resistance	82.0	s m^{-1}
Stomatal	Le	Lewis number	1.47	—
Stomatal	lf	Leaf characteristic length	0.1	m
Stomatal	va	Air velocity	0.09	m s^{-1}
Stomatal	rt	Transfer resistance	50.0	s m^{-1}
Stomatal	c_rc_1	Cuticular resistance coeff. 1	0.315	—
Stomatal	c_rc_2	Cuticular resistance coeff. 2	−27.35	—
Stomatal	c_rc_3	Cuticular resistance coeff. 3	790.7	—
CO_2 transport	rho_CO2_T0	CO_2 density at T0	1.98	kg m^{-3}

> *Note: The c_alpha parameter in the UV-A extension layer (0.54) overrides the Sun model's internal value (0.68) to calibrate for the specific LED spectrum used in our experiments.*

Table S8 Environment Conditions for Simulation

Growth chamber settings used for all simulations.

Parameter	Day Value	Night Value	Unit
Photoperiod	6:00–22:00 (16 h)	22:00–6:00 (8 h)	—
Temperature (T)	25	18	°C
Relative humidity (RH)	0.70	0.85	—
CO_2 concentration	1200	1200	ppm
PPFD	130	0	µmol m^{-2} s^{-1}

Parameter	Day Value	Night Value	Unit
Baseline irradiance (I_base)	57	0	W m^{-2}
UV-A irradiance (I_UVA)	1,100 (when applied)	0 or 1,100 (night treatment)	μW cm^{-2}
Plant density	36	36	plants m^{-2}

> *Note: UV-A treatments were applied during the light period (daytime) except for the L6D6-N treatment, which applied UV-A during the dark period (nighttime) to test circadian effects. The higher nighttime RH (0.85) reflects natural humidity increase due to reduced transpiration and temperature drop.*

Table S9 Conceptual Parameters in Main Text Equations

Parameter	Description	Implementation
K_carbon	Half-saturation constant for carbon-limited growth	Embedded in Sun model's C_buf dynamics. h_buf feedback operates as limiter: h_buf = 1.0 when C_buf < C_buf_max; when C_buf ≥ C_buf_max, h_buf = min[(R_d + RGR_max × X_d / c_beta) / (c_alpha × A_C), 1.0]. Here C_buf_max = σ_buf × X_d where σ_buf = 0.2.
k_senescence	LAI senescence/turnover rate	Incorporated in Sun model's leaf area dynamics; accounts for natural leaf turnover through shoot:root allocation and the relationship dLAI/dt = SLA × dX_d_shoot/dt, where X_d_shoot is determined by the dynamic shoot:root ratio σ_r(T, RH).

Table S10 Main Text Symbol ↔ Code Variable Correspondence

Math Symbol	Code Variable	Equation	Description
α_SLA	uva_sla_enhancement	Eq.(5)	Maximum UV-A enhancement on SLA
K_SLA	K_uva_sla	Eq.(5)	Half-saturation UV-A intensity for SLA enhancement
α_LAI	uva_lai_boost	Eq.(5)	Maximum UV-A boost on LAI growth
K_LAI	K_uva_lai	Eq.(5)	Half-saturation UV-A intensity for LAI boost
k_prod	k_ros_production	Eq.(6)	ROS production coefficient
k_clear	k_ros_clearance	Eq.(6)	ROS clearance coefficient
stress_coeff	stress_damage_coeff	Eq.(7)	Vulnerability damage coefficient ($1.6×10^{-7}$)
k_nonlin	k_nonlinear_stress	Eq.(7)	Nonlinear damage coefficient ($5.0×10^{-6}$)
A_vuln	A_vulnerability	Eq.(7)	Amplitude of LAI-dependent vulnerability
k_vuln	k_vulnerability	Eq.(7)	Decay of vulnerability with LAI
k_decay	k_stress_decay	Eq.(7)	Stress decay rate ($2.14×10^{-5}$ s^{-1})
F_max	gompertz_max_factor	Eq.(7)	Maximum Gompertz damage amplification

Math Symbol	Code Variable	Equation	Description
H_threshold	gompertz_threshold	Eq.(7)	Gompertz inflection point (hours)
k_steep	gompertz_steepness	Eq.(7)	Gompertz steepness
α_prot	alpha_aox_protection	Eq.(7)	Maximum AOX protection efficiency
K_prot	K_aox_protection	Eq.(7)	Half-saturation for AOX protection
k_circ	k_circadian	Eq.(7)	Circadian damage coefficient
n_circ	n_circadian	Eq.(7)	Exponent for circadian damage
S_AOX	aox_synthesis_rate	Eq.(3,8)	Total AOX synthesis rate
C_cost	aox_carbon_cost	Eq.(3)	Carbon cost per AOX synthesized
CC_penalty	carbon_competition_effect	Eq.(4)	Carbon competition penalty on growth
Stress_inhib (X_d)	stress_photosynthesis_inhibition	Eq.(4)	Maximum stress inhibition on dX_d/dt (0.85)
Stress_inhib (LAI)	stress_lai_inhibition	Eq.(4)	Maximum stress inhibition on dLAI/dt (0.80)
K_stress	K_stress	Eq.(4)	Half-saturation for stress inhibition (50.0)

Table S11 Numerical Integration Method

ODE system solved using scipy.integrate.solve_ivp.

Parameter	Value	Description
Method	RK45 (Dormand-Prince)	Explicit Runge-Kutta method of order 5(4)
Relative tolerance (rtol)	1×10^{-3}	scipy default
Absolute tolerance (atol)	1×10^{-6}	scipy default
Maximum step size	300 s	Prevents large jumps during day/night transitions
Time step	Adaptive	Automatically adjusted by solver
Output points	100 (500 for figures)	Uniformly spaced; 500 used in generate_paper_figures.py for smoother visualization
Simulation period	14–35 DAS	21 days (1,814,400 seconds)

Table S12 Sun Model Core ODE Equations

Three-state ODE system from Sun et al. (2025), serving as foundation for the UV-A extension.

State Variable 1: Structural Dry Weight (X_d)

$$dX\_d/dt = c\_beta \times (c\_alpha \times A\_C \times h\_buf - R\_d) \quad (S6)$$

State Variable 2: Carbon Buffer (C_buf)

$$dC\_buf/dt = (c\_alpha \times A\_C \times h\_buf - R\_d) - (RGR\_max \times X\_d / c\_beta) \quad (S7)$$

State Variable 3: Leaf Area Index (LAI)

dLAI/dt = dX_d/dt × (1 − σ_r) × SLA (S8)

Key Auxiliary Variables

Variable	Formula	Eq.
h_buf (normal)	h_buf = 1.0 if C_buf < C_buf_max h_buf = min[(R_d + RGR_max×X_d/c_beta)/(c_alpha×A_C), 1.0] if C_buf ≥ C_buf_max	S9
R_d (dark resp.)	[c_Rd_25_sh×(1−σ_r) + c_Rd_25_r×σ_r] × X_d × Q10_Rd^((T_c−25)/10)	S10
RGR_max	RGR_max_20 × Q10_gr^((T_c−20)/10) if T_c ≤ T_c_RGR RGR_max_20 × Q10_gr^(−(T_c−20)/10) if T_c > T_c_RGR	S11
σ_r	clip[c_σr_1 × ln(plant_DW) + c_σr_2, 0.05, 0.35] where plant_DW = X_d / plant_density	S12
SLA	SLA_ref × f_I_SLA × f_Xh_SLA	S13
A_C	A_L_C × LAI (Gaussian quadrature across canopy layers)	S14
A_L	A_L_sat × [1 − exp(−ε × PAR_a / A_L_sat)]	S15

UV-A Extension Modifications to Sun Model

#	Modification
1	Add three state variables: AOX, Stress, ROS
2	Multiply dX_d/dt by (1 − Stress_inhib) × (1 − CC_penalty)
3	Subtract AOX carbon consumption from dC_buf/dt
4	Replace SLA with SLA_new in the LAI equation for UV-A morphological effects

Table S13 Stomatal Conductance & Resistance Formulas

Total CO_2 Resistance (S16)

r_CO2 = r_s + r_b + r_c + r_t

Stomatal Resistance (S17)

r_s = c_zeta × r_H2O_min × f_I × f_T × f_Xc × f_Xh

Response Function	Formula
f_I (irradiance)	(I_a/(2×LAI) + 4.3) / (I_a/(2×LAI) + 0.54)
f_T (temp., light)	1 + 0.023×(T_c − 24.5)^2 if I > 3 W m^{-2}
f_T (temp., dark)	1 + 0.005×(T_c − 33.6)^2 if I ≤ 3 W m^{-2}
f_Xc (CO_2)	1 + 6.1×10^{-7}×(X_c − 200)^2 if I > 3 and X_c < 1100
f_Xh (humidity)	4.0 / (1 + 255×exp(−0.0054×e_c))^0.25

Boundary Layer Resistance (S18)

r_b = Le^0.67 × 1174 × l_f^0.5 / (l_f×∣T_leaf−T_air∣ + 207×v_a^2)^0.25

Cuticular Resistance (S19)

$r\_c = \max(0.315 \times T\_c^2 - 27.35 \times T\_c + 790.7, 10.0)$

Light-Saturated Assimilation (S20)

$A\_L\_cn = \varrho\_CO2 \times (X\_c - \Gamma) / r\_CO2 \times 10^{-6}$

$A\_L\_mm = M\_CO2 \times J\_max / 4 \times 10^{-6}$

$A\_L\_sat\_n = \min(A\_L\_cn, A\_L\_mm)$

$A\_L\_sat = \max(A\_L\_sat\_n + R\_d / (LAI \times c\_alpha), 0)$

Table S14 Energy Balance and Leaf Temperature

The Sun model uses a simplified energy balance approach where leaf temperature is assumed to equal air temperature (T_leaf ≈ T_air) for the boundary layer resistance calculation.

Justification

In controlled-environment growth chambers with forced air circulation ($v\_a = 0.09$ m s^{-1}), the leaf-to-air temperature difference is typically small (<2°C). The model uses air temperature directly for:

photosynthetic rate calculations (J_max temperature response),
respiration rate calculations (Q_10 relationships), and
stomatal conductance temperature response.

Implementation Note

The term |T_leaf − T_air| in the boundary layer resistance formula is set to a small positive value to avoid division by zero while maintaining the forced convection term ($207 \times v\_a^2$). For applications requiring explicit leaf temperature calculation, the full energy balance equation would be:

$$R\_n = H + \lambda E + G$$

where R_n is net radiation, H is sensible heat flux, λE is latent heat flux, and G is heat storage (negligible for leaves).

Table S15 Complete AOX Synthesis Formula

Equation S21 — Total AOX Synthesis Rate

$$S\_AOX = LAI \times (S\_base + S\_UV + S\_stress \times f\_adapt \times f\_nonlin) \times f\_stress\_synth \times f\_water \times f\_carbon$$

Component	Formula
S_base (baseline)	base_aox_rate_light (day) or base_aox_rate_dark (night)
S_UV (UV-induced)	k_uv_aox × total_uva_hours / (K_uv_hours + total_uva_hours) total_uva_hours = (days_irradiated−1)×daily_hours + hours_today
S_stress (stress-induced)	V_max_aox × Stress / (K_stress_aox + Stress) × f_night × f_LAI f_night = 0.4 for night-irradiation treatments, else 1.0 f_LAI = min(1.0, (LAI/LAI_healthy)^n_LAI_eff)
f_adapt (adaptation)	K_adapt_days / (K_adapt_days + days_irradiated) Reflects decreasing induction efficiency with prolonged exposure
f_nonlin (nonlinear eff.)	1 / (1 + (Nonlinear_Factor / K_nonlin_aox)^n_nonlin_aox) Hill-type inhibition based on Gompertz damage factor

Component	Formula
f_stress_synth	1 − max_stress_inhib × Stress^n / (K_stress_inhib^n + Stress^n)
f_water	1.0 if LDMC ≤ threshold Else: 1 − water_aox_max_inhib × (LDMC−thr)^n / (water_aox_K^n + (LDMC−thr)^n)
f_carbon	1 − 0.20 × carbon_competition_effect

Table S16 AOX Consumption Formula

Equation S22

$$C\_AOX = k\_aox\_consumption \times f\_amp \times AOX \times ROS^n / (K\_ros\_consumption^n + ROS^n)$$

Consumption Amplification (f_amp) — Softplus Activation

$$x\_raw = (Nonlinear\_Factor - cons\_amp\_center) / cons\_amp\_scale$$

$$x = cons\_amp\_scale \times \ln(1 + \exp(x\_raw))$$

$$f\_amp = 1 + cons\_amp\_k \times x^2 / (cons\_amp\_K^2 + x^2)$$

Note: Amplification reflects accelerated AOX turnover when daily exposure exceeds the damage threshold (~10.5 h), consistent with increased ROS load overwhelming antioxidant capacity.

Table S17 LDMC Dynamics Formula

Equation S23 — Dynamic LDMC Calculation

$$LDMC = \min(dw\_fw\_ratio\_base \times (1 + stress\_effect \times acute\_factor), dw\_fw\_ratio\_max)$$

$$stress\_effect = ldmc\_stress\_sensitivity \times Stress / (K\_ldmc + Stress)$$

Acute Factor (Softplus Activation)

$$x\_raw = (Nonlinear\_Factor - acute\_center) / acute\_scale$$

$$x = acute\_scale \times \ln(1 + \exp(x\_raw))$$

$$acute\_factor = 1 + acute\_k \times x^{acute\_n} / (acute\_K^{acute\_n} + x^{acute\_n})$$

Note: The acute factor amplifies LDMC increase when Nonlinear_Factor > 50, representing rapid dehydration under prolonged UV-A stress.

Table S18 UV-A Morphological Effects with Stress Suppression

Equation S24 — Stress Suppression Factor

$$stress\_suppression = 1 - Stress / (K\_stress + Stress)$$

Equation S25 — Modified Morphological Boosts

$$SLA\_boost\_effective = \alpha\_SLA \times I\_UVA / (K\_SLA + I\_UVA) \times stress\_suppression \quad (S25a)$$

$$LAI\_boost\_effective = \alpha\_LAI \times I\_UVA / (K\_LAI + I\_UVA) \times stress\_suppression \quad (S25b)$$

Condition	dX_d/dt Modifier	dLAI/dt Modifier
Positive growth	× (1 + SLA_boost_effective × 0.5)	× (1 + LAI_boost_effective)

Condition	dX_d/dt Modifier	dLAI/dt Modifier
Negative growth (senescence)	× (1 − SLA_boost_effective × 0.15)	× (1 − LAI_boost_effective × 0.3)

> *Note: Stress suppression reflects that plants under severe oxidative stress prioritize survival over morphological optimization, consistent with the Growth-Differentiation Balance Hypothesis.*

Supplementary Figures

Supplementary Figure S1. System dynamics block diagram. Block diagram illustrating the six-state ODE model structure with carbon competition mechanism. Shows: (1) environmental inputs (PAR, UV-A, temperature); (2) the six state variables (X_d, C_buf, LAI, AOX, Stress, ROS); (3) key feedback loops including ROS→Stress→Growth inhibition, AOX protection of damage, and carbon competition between growth and AOX synthesis; (4) the carbon buffer pool connecting photosynthesis, growth, and defense metabolism.

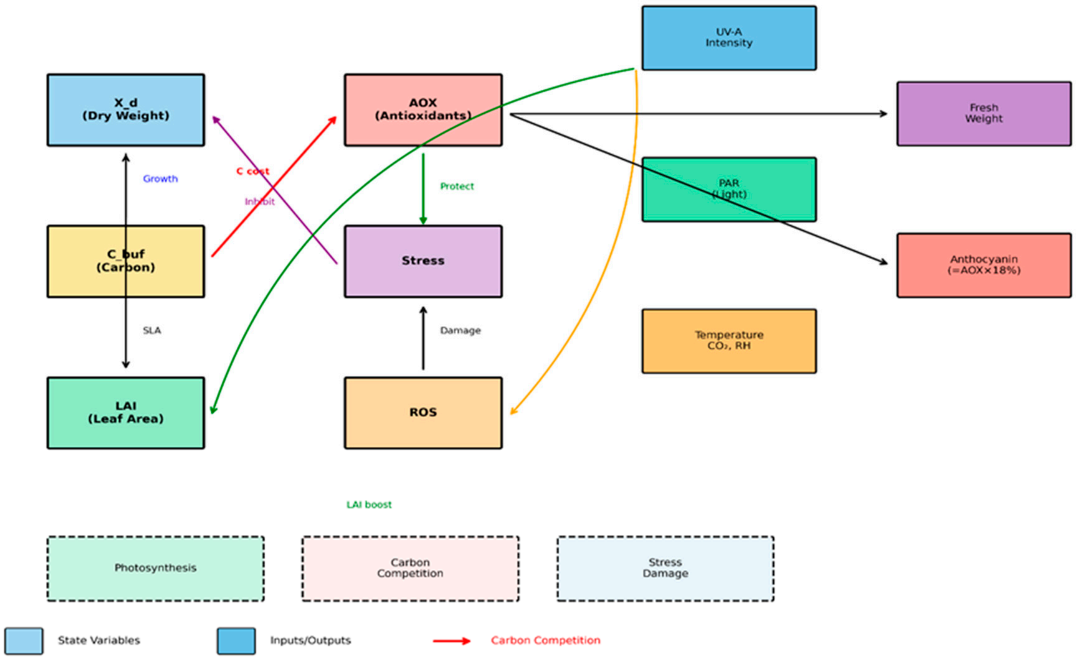

Supplementary Figure S2. Hormesis response surface. Three-dimensional surface plot showing predicted anthocyanin concentration (ppm) as a function of daily UV-A hours (1–12 h day^{-1}) and total treatment days (2–12 d). The surface demonstrates the hormesis phenomenon: anthocyanin initially increases with UV-A dose but declines at extreme doses. The optimal region lies at moderate daily hours (8–10 h) with shorter duration (3–5 d).

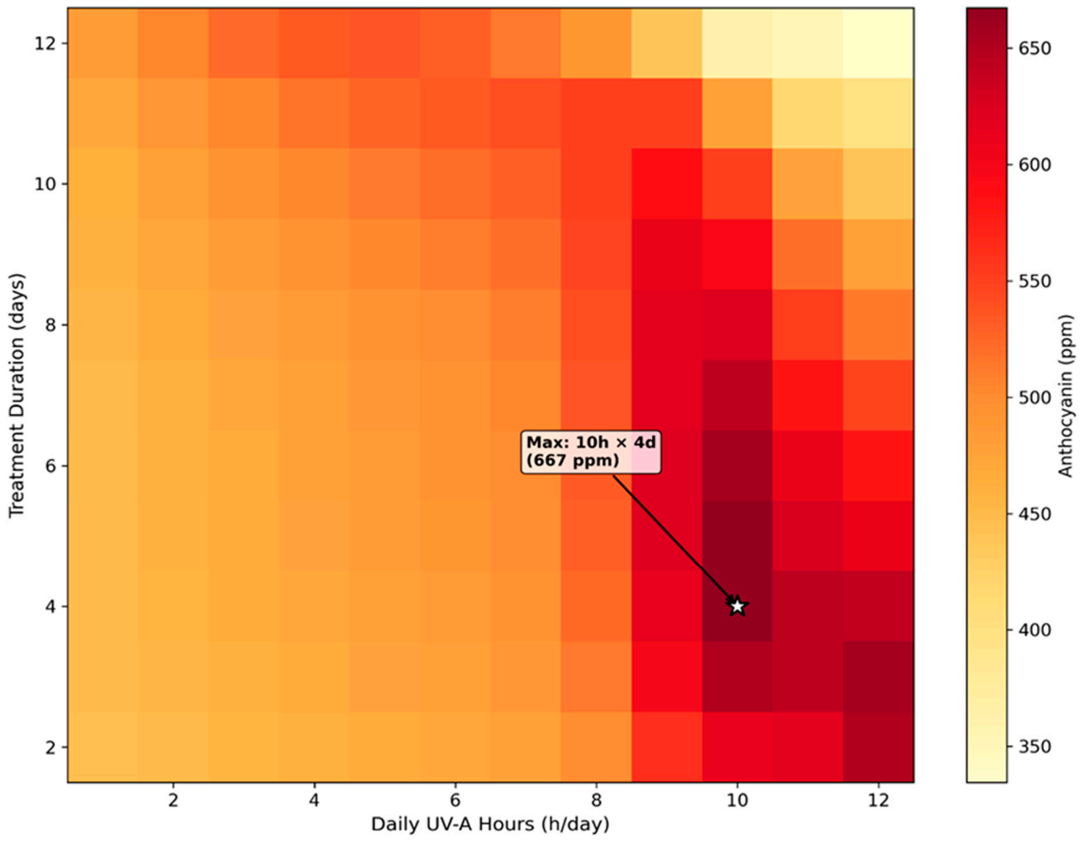

Supplementary Figure S3. Carbon competition mechanism visualization. Multi-panel figure: (a) Carbon buffer (C_buf) dynamics over time showing depletion under high-stress treatments; (b) AOX accumulation trajectories; (c) Carbon competition penalty as a function of stress level; (d) Growth penalty comparison across treatments, consistent with the Growth-Differentiation Balance Hypothesis.

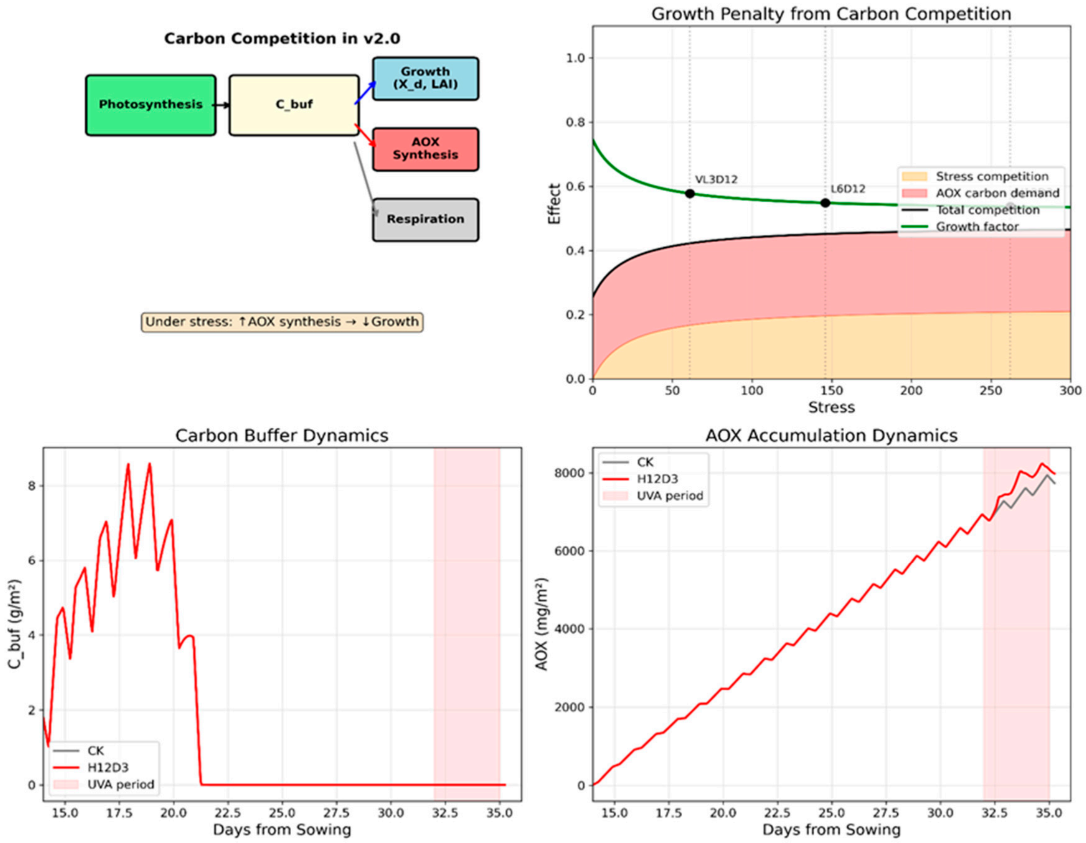
Carbon Competition in v2.0
Photosynthesis
C_buf
Growth (X_d, LAI)
AOX Synthesis
Respiration
Under stress: ↑AOX synthesis → ↓Growth
Growth Penalty from Carbon Competition
Effect
Stress
VL3D12
L6D12
Stress competition
AOX carbon demand
Total competition
Growth factor
Carbon Buffer Dynamics
C_buf (g/m²)
Days from Sowing
CK
H12D3
UVA period
AOX Accumulation Dynamics
AOX (mg/m²)
Days from Sowing
CK
H12D3
UVA period

Supplementary Note S1. Experimental Light Sources and Cultivation Setup

This section provides detailed specifications for all light sources used in this study, including manufacturer information, spectral characteristics, and spatial irradiance distributions.

S1.1 Primary Cultivation Lighting

Primary illumination was provided by T8 LED tubes (Epistar Corporation, Hsinchu, Taiwan) with a correlated color temperature of 4000 K. The spectral composition comprised approximately 30% red, 45% green, and 25% blue wavelengths, delivering a photosynthetic photon flux density (PPFD) of 130 μmol m^{-2} s^{-1} at the canopy level. The corresponding photosynthetically active radiation (PAR) power was approximately 28.5 W m^{-2}, calculated using a spectrum-weighted conversion coefficient $\kappa \approx 4.56$ μmol J^{-1} based on the measured spectral distribution (see Supplementary Figure S4a).

S1.2 UV-A Light Source

UV-A irradiation was provided by fluorescent insect-attracting lamps (TOA Lighting Co., Ltd., Taiwan). These lamps emit primarily in the UV-A range (315–400 nm) with a peak wavelength of 365 nm. The measured irradiance at the canopy level was approximately 1,100 μW cm^{-2}. The spectral emission profile is shown in Supplementary Figure S6a, confirming that the output is confined to the UV-A waveband with negligible emission outside the 340–400 nm range. The spatial irradiance distribution across the cultivation area is shown in Supplementary Figure S7a, demonstrating uniform coverage (coefficient of variation $< 15\%$) over the plant growth zone.

S1.3 UV-B Light Source

UV-B irradiation was provided by germicidal lamps (Sankyo Denki Co., Ltd., Kanagawa, Japan). Although commonly classified as germicidal lamps, these particular lamps have a peak emission wavelength of 302 nm, placing the primary output in the UV-B range (280–315 nm). The measured irradiance at the canopy level was approximately 1,500 μW cm^{-2}. The spectral emission profile (Supplementary Figure S6b) shows that the dominant emission is centered at 302 nm with a minor secondary emission peak near 254 nm in the UV-C range.

Table S19 Summary of Light Source Specifications

Light Source	Manufacturer	Type	Peak λ (nm)	Waveband	Irradiance at Canopy	Application
T8 LED tube	Epistar (Hsinchu, Taiwan)	LED, 4000 K	Broadband (R/G/B)	PAR (400–700 nm)	130 μmol m^{-2} s^{-1}	Primary lighting
Insect-attracting lamp	TOA Lighting (Taiwan)	Fluorescent	365	UV-A (315–400 nm)	~1,100 μW cm^{-2}	UV-A treatment
Germicidal lamp	Sankyo Denki (Japan)	Mercury vapor	302	UV-B (280–315 nm)	~1,500 μW cm^{-2}	UV-B treatment (Stage 1)

S1.4 Cultivation Shelf Configuration

Plants were grown on multi-tier cultivation shelves within a controlled-environment growth chamber. Each shelf tier was equipped with T8 LED tubes mounted at a fixed height above the canopy. For UV treatments, UV-A or UV-B lamps were installed alongside the LED tubes on the same tier, ensuring consistent irradiance across all plants within each treatment group.

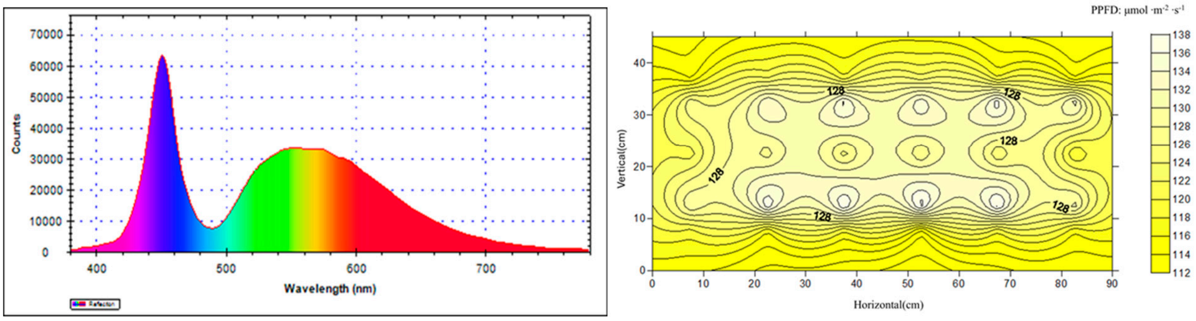

(a) *(b)*

Supplementary Figure S4. Primary LED light source characterization. (a) Spectral distribution of the T8 LED tubes (Epistar, 4000 K) used for primary cultivation lighting, showing emission peaks in the blue (~450 nm), green (~550 nm), and red (~630 nm) regions with relative intensities of approximately 25%, 45%, and 30%. (b) Spatial irradiance distribution (PPFD, μmol m^{-2} s^{-1}) measured at the canopy level.

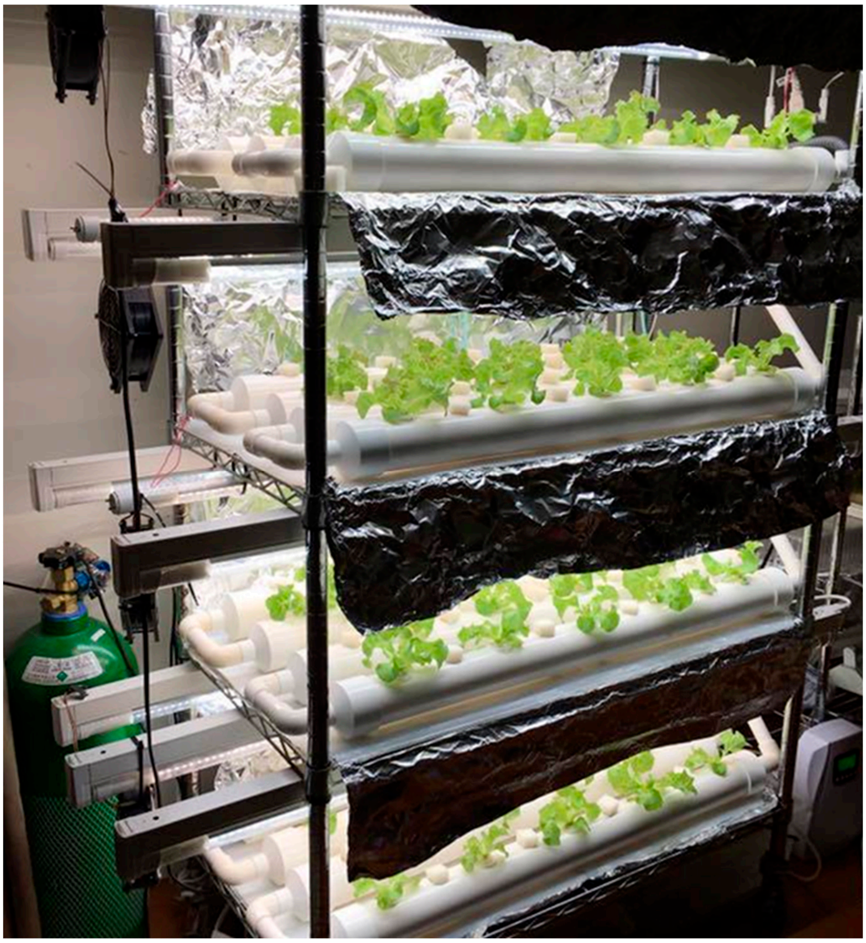

(a)

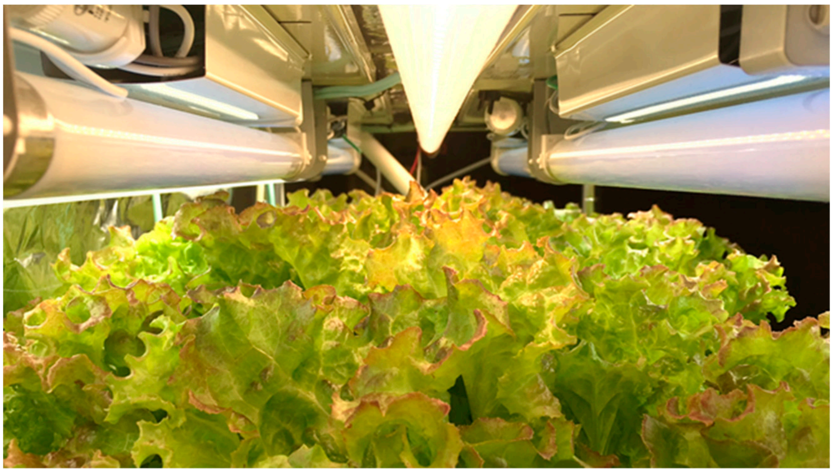

(b)

Supplementary Figure S5. Cultivation shelf and lamp configuration. (a) Photograph of the multi-tier cultivation shelf system within the growth chamber. (b) Close-up showing lamp configuration on a single tier, illustrating the positioning of T8 LED tubes and supplementary UV lamps.

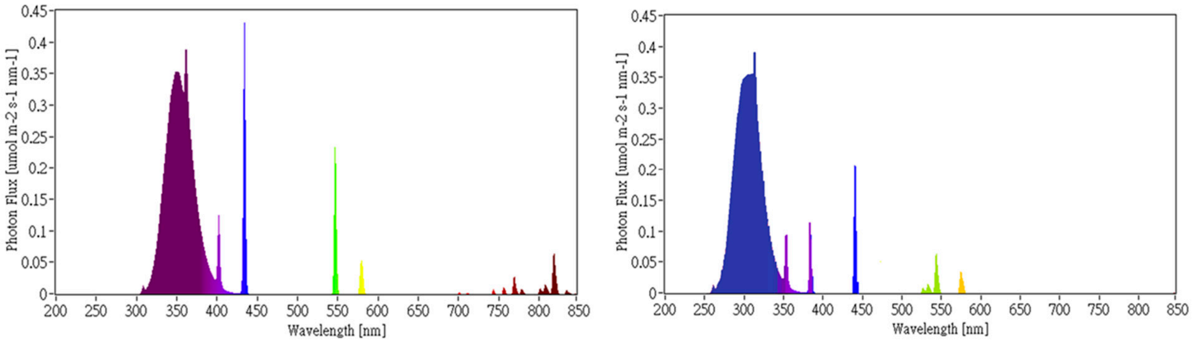

(a) (b)

Supplementary Figure S6. UV lamp spectral distributions. (a) Spectral emission profile of the UV-A fluorescent insect-attracting lamp (TOA Lighting), showing a dominant emission peak at 365 nm within the UV-A range. (b) Spectral emission profile of the UV-B germicidal lamp (Sankyo Denki), showing a primary emission peak at 302 nm with a minor secondary emission line near 254 nm (UV-C).

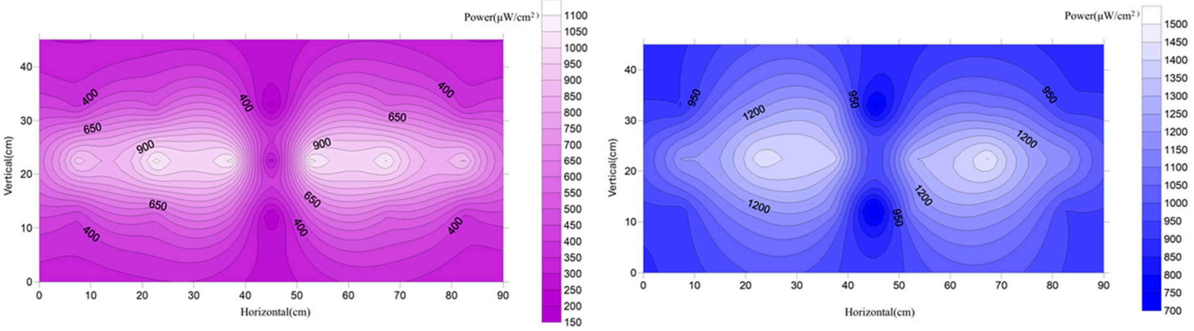

(a)

Supplementary Figure S7. UV lamp spatial irradiance distributions. (a) Spatial irradiance distribution (μW cm^{-2}) of the UV-A lamp at canopy level. (b) Spatial irradiance distribution (μW cm^{-2}) of the UV-B lamp at canopy level.

Code Availability

Full implementation, configuration files and validation scripts are available at:

https://github.com/GrayWei444/uva-simulation

File	Purpose
lettuce_uva_model.py	Main simulation code with carbon competition mechanism
lettuce_uva_carbon_complete_model.py	Base Sun model (dependency)
parameters.md	Complete parameter documentation
generate_paper_figures.py	Script to generate all figures (S1–S3)

> *Note: We recommend citing a tagged release and archiving via Zenodo to obtain a DOI for peer-review reproducibility.*
